# A Multiplex Two-Color Real-Time PCR Method for Quality-Controlled Molecular Diagnostic Testing of FFPE Samples

**DOI:** 10.1371/journal.pone.0089395

**Published:** 2014-02-21

**Authors:** Jiyoun Yeo, Erin L. Crawford, Thomas M. Blomquist, Lauren M. Stanoszek, Rachel E. Dannemiller, Jill Zyrek, Luis E. De Las Casas, Sadik A. Khuder, James C. Willey

**Affiliations:** 1 Division of Pulmonary/Critical Care and Sleep Medicine, Department of Medicine, University of Toledo Health Sciences Campus, Toledo, Ohio, United States of America; 2 Department of Pathology, University of Toledo Health Sciences Campus, Toledo, Ohio, United States of America; University of Houston, United States of America

## Abstract

**Background:**

Reverse transcription quantitative real-time PCR (RT-qPCR) tests support personalized cancer treatment through more clinically meaningful diagnosis. However, samples obtained through standard clinical pathology procedures are formalin-fixed, paraffin-embedded (FFPE) and yield small samples with low integrity RNA containing PCR interfering substances. RT-qPCR tests able to assess FFPE samples with quality control and inter-laboratory reproducibility are needed.

**Methods:**

We developed an RT-qPCR method by which 1) each gene was measured relative to a known number of its respective competitive internal standard molecules to control for interfering substances, 2) two-color fluorometric hydrolysis probes enabled analysis on a real-time platform, 3) external standards controlled for variation in probe fluorescence intensity, and 4) pre-amplification maximized signal from FFPE RNA samples. Reagents were developed for four genes comprised by a previously reported lung cancer diagnostic test (LCDT) then subjected to analytical validation using synthetic native templates as test articles to assess linearity, signal-to-analyte response, lower detection threshold, imprecision and accuracy. Fitness of this method and these reagents for clinical testing was assessed in FFPE normal (N = 10) and malignant (N = 10) lung samples.

**Results:**

Reagents for each of four genes, *MYC, E2F1, CDKN1A* and *ACTB* comprised by the LCDT had acceptable linearity (R^2^>0.99), signal-to-analyte response (slope 1.0±0.05), lower detection threshold (<10 molecules) and imprecision (CV <20%). Poisson analysis confirmed accuracy of internal standard concentrations. Internal standards controlled for experimentally introduced interference, prevented false-negatives and enabled pre-amplification to increase signal without altering measured values. In the fitness for purpose testing of this two-color fluorometric LCDT using surgical FFPE samples, the diagnostic accuracy was 93% which was similar to that previously reported for analysis of fresh samples.

**Conclusions:**

This quality-controlled two-color fluorometric RT-qPCR approach will facilitate the development of reliable, robust RT-qPCR-based molecular diagnostic tests in FFPE clinical samples.

## Introduction

Reverse transcription quantitative real-time polymerase chain reaction (RT-qPCR) tests that measure transcript abundance of selected genes in clinical specimens have been demonstrated to increase cancer diagnostic accuracy and enable “personalized medicine” through selection of the most effective treatment for each cancer [1]–[3]. However, a key challenge is that current clinical pathology sample collection and processing procedures focus on formalin fixation and paraffin embedding (FFPE) and fresh/fresh-frozen tissues rarely are available for molecular analysis. FFPE samples are difficult to work with because they yield RNA that 1) often contains PCR-interfering substances, and 2) is uniformly highly fragmented and often in low abundance [4]–[8]. Economic factors prevent changing this workflow to ensure collection of samples in a form more conducive to molecular genetic analysis, such as fresh frozen, therefore, there is a need to develop methods that are sufficiently robust to reliably conduct molecular genetic analysis in FFPE samples.

One way to address the challenge of interfering substances is to incorporate quality control in qPCR through measurement of each analyte relative to a known number of competitive template internal standard (IS) copies. This quality control method is recommended by regulatory agencies, including the EPA [9], ISO [10], and FDA [11], and is implemented in many FDA-approved single analyte RT-qPCR tests [12]–[14].

Multiple gene RT-qPCR tests present an even more complex quality control challenge that can be addressed by combining the IS for each of the genes into an internal standards mixture (ISM) and using an aliquot of this ISM in each experiment, as previously described [15]–[17]. Each target gene and loading control gene then is measured relative to a known number of its respective competitive template IS molecules in each PCR reaction [18], [19].

To address the challenge of analyzing clinical samples that yield a low amount of RNA, we previously described a competitive multiplex PCR method, in which all reference and target genes are first co-amplified with ISM in a first round of PCR, followed by amplification of individual gene in the second round [20]. This approach enables reliable measurement of many genes from the amount of RNA that, without pre-amplification, would be sufficient for measurement of only a single gene.

The primary goal of this study was to develop a robust RT-qPCR method for more reliable molecular diagnostic testing of FFPE samples including those stored in existing large archives. To meet this need, a method was designed with four elements: 1) Synthetic competitive IS formulated into an ISM to control for sub-optimal PCR, including interference with PCR caused by substances present in FFPE samples [21]–[23], sub-optimal quantity or quality of PCR reaction reagents, and inter-well and/or inter-platform variation in PCR conditions; 2) Fluorometric hydrolysis probe real-time PCR to enable quantification of short PCR amplicons (60–80 base pairs) that are optimal for reliable analysis of FFPE samples; 3) An external standards mixture (ESM) in each experiment to control for inter-lot and inter-experimental variation in probe fluorescence intensity; and 4) Reverse transcription with gene specific primers (GSP) followed by competitive multiplex pre-amplification to enable measurement of lowly expressed genes in pauci-cellular samples with degraded and limited amounts of RNA.

To validate this approach, we developed reagents for measurement of previously reported lung cancer diagnostic test (LCDT) comprising v-*myc* avian myelocytomatosis viral oncogene homolog (*MYC),* E2F transcription factor 1 *(E2F1)*, and cyclin-dependent kinase inhibitor 1A (*CDKN1A)* genes measured relative to actin, beta (*ACTB)* [24]. These reagents were subjected to analytical validation using synthetic templates as test articles and fitness for the purpose of testing using surgical benign and malignant FFPE samples according to recommended practices [25].

## Materials and Methods

### Ethics Statement

Twenty archived surgical FFPE lung tissues that had been processed according to the standard University of Toledo Medical Center (UTMC) Department of Pathology practice between 2010 and 2012 were obtained for this study under UTMC IRB # 107790. According to the UTMC IRB # 107790 protocol, each FFPE sample was assigned a non-identifying number by the pathologist and transferred to the research laboratory. The link between the non-identifying number and identifying information was destroyed by the pathologist immediately following sample transfer. Accordingly, on March 27, 2013 the UTMC IRB #107790 protocol was approved by the Chair of the UTMC Biomedical Institutional Review Board as exempt research and the requirement to obtain a signed consent/authorization form for use and disclosure of protected health information was waived as this research was determined to be minimal risk.

### FFPE Sample Preparation

Microtome sections (10 micrometre thickness) were obtained from each sample. Six strips per sample (1 strip = 4 sections) were obtained, and each strip was put in one 1.5 ml micro-centrifuge tube for RNA extraction. Therefore, 24 sections (240 micrometres) of each sample block were collected for RNA extraction.

### RNA Extraction and Reverse Transcription

RNA was extracted from the surgical FFPE samples using the RNeasy FFPE Kit (Qiagen, Valencia, CA). RNA was treated with DNase in the RNeasy FFPE Kit RNA extraction protocol in order to minimize the effect of contaminating genomic DNA. RNA purity and integrity were assessed using absorbance at 260/280 nm ratios and RNA integrity number (RIN) scores as detected on the Agilent 2100 Bioanalyzer (Agilent Technologies, Santa Clara, CA). The effect of different conditions on reverse transcription (RT) efficiency, including priming with random hexamer primers (RHP) or GSP and use of 1 or 5 µg of RNA in the 30 µl RT reaction, was assessed with three FFPE samples. A previously described test using the External RNA Control Consortium (ERCC) standards was used to measure RT efficiency [26]. After completion of these studies, optimal RT conditions were selected consisting of a 30 µl RT reaction with 1 µg of RNA, gene-specific RT primer (the PCR reverse primer) (3 µM), and SuperScript III First Strand Synthesis System (Life Technologies, Grand Island, NY).

### Primer Design and Testing of Efficiency and Specificity

For each gene (*MYC, E2F1, CDKN1A* and *ACTB*) primers were designed to 1) amplify the shortest possible PCR product size (60–80 base pairs) and 2) span intron/exon splice junctions to minimize the effect of residual genomic DNA contamination (Table 1).

**Table 1:** Sequences of primers, probes, and standard templates for two-color fluorometric real-time measurement.

Primer sequences					
Gene	Location	GenBank accession no.	Primer	Sequence (5′- 3′)	Size
*ACTB*	Exon 4, 5	NM_001101.3	Forward	GCCCTGAGGCACTCTTCCAG	20 bp
			Reverse	TTTCGTGGATGCCACAGGAC	20 bp
*CDKN1A*	Exon 5, 6	NM_000389.4	Forward	CCTGGAGACTCTCAGGGTCG	20 bp
			Reverse	GCGTTTGGAGTGGTAGAAAT	20 bp
*MYC*	Exon 1, 2	NM_002467.4	Forward	AGCTGCTTAGACGCTGGATT	20 bp
			Reverse	CTAACGTTGAGGGGCATCGT	20 bp
*E2F1*	Exon 5, 6	NM_005225.2	Forward	CTCCTCAGGGCACAGGAA	18 bp
			Reverse	CGTGGACTCTTCGGAGAACTTTC	23 bp
**Probe sequences**					
**Gene**	**Probe**	**Modification 5′**	**3′**	**Sequence (5′- 3′)**	**Size**
*ACTB*	NT probe	FAM	BHQ *plus*-1	CCTTCCTTCCTGGGCATG	18 bp
	IS probe	Quasar 670	BHQ *plus*-2	CC***AA***CCTTCC***A***GGGCAT***C***	18 bp
*CDKN1A*	NT probe	FAM	BHQ *plus*-1	AAACGGCGGCAGACCAGC	18 bp
	IS probe	Quasar 670	BHQ *plus*-2	***TT*** ACGGCGG***GT***GACCA***C***	17 bp
*MYC*	NT probe	FAM	BHQ *plus*-1	TAGTGGAAAACCAGCAGCCT	20 bp
	IS probe	Quasar 670	BHQ *plus*-2	***AT*** GTGGAAA***T***CC***T***GCAGC***GA***	20 bp
*E2F1*	NT probe	FAM	BHQ *plus*-1	CATCGATCGGGCCTTGTT	18 bp
	IS probe	Quasar 670	BHQ *plus*-2	***TTC*** CGATCG***T***GCCTT***C***T***A***	18 bp
**NT and IS sequences**					
**Gene**	**Template**	**Sequence (5′- 3′)**			**Size**
*ACTB*	NT	GCCCTGAGGCACTCTTCCAGCCTTCCTTCCTGGGCATGG AGTCCTGTGGCATCCACGAAA			60 bp
	IS	GCCCTGAGGCACTCTTCCAGCC***AA***CCTTCC***A***GGGCAT***C***G AGTCCTGTGGCATCCACGAAA			60 bp
*CDKN1A*	NT	CCTGGAGACTCTCAGGGTCGAAAACGGCGGCAGACCAGC ATGACAGATTTCTACCACTCCAAACGC			66 bp
	IS	CCTGGAGACTCTCAGGGTCGA***TT*** ACGGCGG***GT***GACCA***C***C ATGACAGATTTCTACCACTCCAAACGC			66 bp
*MYC*	NT	AGCTGCTTAGACGCTGGATTTTTTTCGGGTAGTGGAAAA CCAGCAGCCTCCCGCGACGATGCCCCTCAACGTTAG			75 bp
	IS	AGCTGCTTAGACGCTGGATTTTTTTCGGG***AT*** GTGGAAA***T*** CC***T***GCAGC***GA***CCCGCGACGATGCCCCTCAACGTTAG			75 bp
*E2F1*	NT	CTCCTCAGGGCACAGGAAAACATCGATCGGGCCTTGTTT GCTCTTAAGGGAGATCTGAAAGTTCTCCGAAGAGTCCACG			79 bp
	IS	CTCCTCAGGGCACAGGAAAA***TTC*** CGATCG***T***GCCTT***C***T***A***T GCTCTTAAGGGAGATCTGAAAGTTCTCCGAAGAGTCCACG			79 bp

NT: native template. IS: internal standard. BHQ: black hole quencher. Bold: modified nucleotides. Underline: probe binding site.

Each candidate primer pair was assessed for efficiency in a serially diluted mixture of H23 cell line cDNA and ISM using endpoint PCR. After 35 PCR cycles, products were electrophoretically separated on an Agilent 2100 Bioanalyzer (Agilent Technologies, Santa Clara, CA), the electropherogram was inspected for presence or absence of non-specific products, and appropriately sized peaks were quantified by densitometry.

### Design of Probes and IS Templates

**Figure 1:**
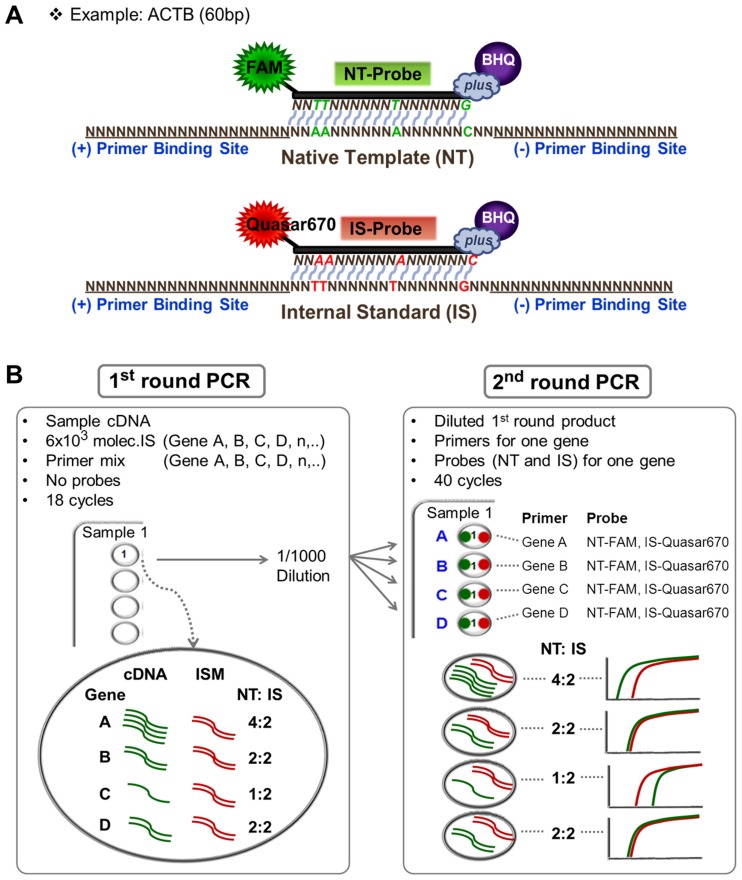
Schematic illustration of the probe design (A) and pre-amplification PCR (B). Native template (NT) binding hydrolysis probes were labeled with FAM. Internal standard (IS) binding hydrolysis probes were labeled with Quasar 670. (A) For each gene, NT and IS had the same primer binding sites but there was a 4–6 bp difference in probe binding sites. (B) Varying concentrations of internal standards mixture (ISM) relative to cDNA were used to ensure that NT: IS ratio was >1∶10 and <10∶1.

For each gene target, after native template (NT) primers with sufficient efficiency and specificity were identified, we developed real-time PCR assays using fluorometric hydrolysis probes [27]. First, a probe for the NT was designed followed by the design of an IS probe for the same DNA region but with 4–6 base pair alterations from the NT probe sequence. An IS template with corresponding alterations was synthesized as described in the synthesis and purification of standards section below. Use of multiple base changes in the IS probe ensured specificity of NT probe (FAM labeled) for the NT and IS probe (Quasar 670 labeled) for the IS. Probes with a fluorescent label at the 5′ end and a Black Hole Quencher at the 3′ end (BHQ*plus*, Biosearch Technologies, Novato, CA) were designed using real-time design software from the Biosearch Technologies web site (Figure 1A, Table 1).

### Synthesis and Purification of Standards

For each gene, we synthesized an NT (to be used in the ESM) and an IS via commercial vendor (Life Technologies, Grand Island, NY). The products of such syntheses are single-stranded and contain a significant fraction of incompletely synthesized (less than full-length) molecules. Thus, we PCR-amplified each synthesized NT or IS with GSP to generate completely synthesized, double-stranded nucleic acid templates. This was followed by electrophoretic separation of the PCR products on agarose gel, selection of the correct size band, and purification from agarose using QIAX II gel extraction kit (Qiagen, Valencia, CA) (Table 1).

### Probe Specificity Test

Specificity of each probe was tested by including it in PCR assays containing the synthetic NT or IS serially diluted from 10^−11^ M to 10^−15^ M. For each probe, at each NT or IS dilution, the signal (Cq value: quantification cycle) [28] observed with amplification in the presence of the non-homologous template was compared to Cq value observed with amplification in the presence of the homologous template. The non-specific binding rate was calculated using 2^(−delta Cq)^ (delta Cq = non-homologous template Cq – homologous template Cq) at each dilution. If, at any concentration, the number of input non-homologous molecules detected by the probe was more than 10% of the number of homologous molecules detected, then the probe was re-designed.

### Preparation of Internal Standards Mixture (ISM)

Known quantities of the IS for each gene were combined into an ISM. Use of the ISM rather than individual IS in each experiment minimized inter-experimental variation [16] as described in Table S1A in File S1. Six different ISM were prepared (ISM A–F) containing different concentrations of target gene IS mixture (*MYC, E2F1, CDKN1A*) relative to the reference gene (*ACTB*) IS.

### External Standards Mixture (ESM)

Known quantities of purified synthetic NT and IS for each gene were combined into an ESM. The ESM was used to control for inter-experimental variation resulting from 1) instability or intensity differences of one fluor relative to the other and 2) software selection of Cq. We prepared a stock ESM with 10^−11^ M NT/10^−11^ M IS, and then diluted it to two working concentrations of 10^−13^ M NT/10^−13^ M IS and 10^−14^ M NT/10^−14^ M IS. Each of these two ESM concentrations was measured in each experiment and for each gene the average measured Cq difference [NT Cq - IS Cq] from the two ESM was used to correct the [NT Cq - IS Cq] value measured in each unknown sample (Table S1B in File S1, Figure S1 in File S1).

### Pre-amplification (1^st^ round PCR)

The pre-amplification reaction for each sample was prepared in a 20 µl volume and included 1) 2 µl primer mixture (*ACTB, MYC, E2F1, CDKN1A*) with concentration of each primer at 800 nM (final concentration of each primer in PCR of 80 nM), 2) 1 µl cDNA sample, 3) 1 µl ISM, and 4) 10 µl TaqMan Universal Master Mix II (No UNG: Uracil N-Glycosylase, Life Technologies, Grand Island, NY) with 6 µl RNase-free water. Probes were not used in the pre-amplification. Cycle parameters were 95°C for 10 min then 18 cycles at 95°C for 15 s and 60°C for 1 min. The ABI 7500 Fast real-time PCR system was used with standard mode (software v2.0.6, Life Technologies).

### Second-round PCR

Pre-amplified PCR products were diluted 1000-fold with TE buffer. A 20 µl reaction was prepared for each gene with 1) 1 µl of diluted pre-amplified product, 2) 2 µl of a primer mixture containing each primer for each gene at 8 µM (final concentration of each primer, 800 nM), 3) 2 µl of 2 µM NT probe and 2 µl of 2 µM IS probe (final concentration of each probe, 200 nM), 4) 10 µl TaqMan Universal Master Mix II (No UNG) with 3 µl RNase-free water, and subjected to 40 cycles of PCR using the same cycle parameters as in the pre-amplification. Automatic threshold was used to determine Cq values (Figure 1B, Figure S1 in File S1).

### Calculation of Gene Expression

To quantify the copy number for each gene NT in a cDNA sample, 1) the [NT Cq - IS Cq]_Sample_ for the unknown sample and the average [NT Cq - IS Cq] of two concentrations of ESM ([NT Cq - IS Cq]_ESM_) were calculated, 2) The corrected delta Cq was calculated as: [NT Cq - IS Cq]_Sample_ - [NT Cq - IS Cq]_ESM_, 3) 2^(−corrected delta Cq)^ was multiplied times the known number of input IS copies in the reaction to obtain the gene NT copy number, and 4) each target gene NT value was normalized to the *ACTB* loading control gene NT value, and presented as target gene NT molecules/10^6^ *ACTB* molecules (Table 2, Table S1B in File S1).

**Table 2:** Effect of external standards mixture (ESM) on quality control.

Without ESM correction												
			*ACTB*				*MYC*					
Sample	Day	ISM	NT Cq	IS Cq	Raw ΔCq	Molecules/Assay	NT Cq	IS Cq	Raw ΔCq	Molecules/Assay	*MYC*/10^6^ *ACTB*	
**SM8**	#1	D(−12/−14)	11.7	13.0	−1.3	1.5E+06	16.7	18.5	−1.8	2.1E+04	1.4E+04	
	#2	D(−12/−14)	11.8	11.3	0.5	4.3E+05	19.7	16.4	3.3	6.0E+02	1.4E+03	
		E(−12/−15)	11.7	11.0	0.7	3.7E+05	18.7	19.0	−0.2	7.1E+02	1.9E+03	
	#3	D(−12/−14)	11.6	10.8	0.8	3.4E+05	19.8	16.9	2.9	8.1E+02	2.4E+03	
		E(−12/−15)	12.2	11.2	1.0	3.0E+05	19.8	20.4	−0.6	9.0E+02	3.0E+03	
	#4	D(−12/−14)	18.0	16.6	1.4	2.3E+05	26.2	24.3	1.9	1.6E+03	7.0E+03	
	#5	D(−12/−14)	12.1	13.6	−1.5	1.7E+06	20.2	19.7	0.5	4.3E+03	2.5E+03	
**CV**						**0.90**				**1.75**	**0.99**	
**With ESM correction**												
**SM8**	#1	D(−12/−14)	−1.3	−2.0	0.7	3.8E+05	−1.8	−3.0	1.2	2.5E+03	6.6E+03	
	#2	D(−12/−14)	0.5	0.0	0.4	4.5E+05	3.3	2.3	1.1	2.9E+03	6.5E+03	
		E(−12/−15)	0.7	0.0	0.6	3.8E+05	−0.2	2.3	−2.5	3.4E+03	8.8E+03	
	#3	D(−12/−14)	0.8	−0.6	1.4	2.3E+05	2.9	1.7	1.2	2.7E+03	1.2E+04	
		E(−12/−15)	1.0	−0.6	1.6	2.0E+05	−0.6	1.7	−2.3	2.9E+03	1.5E+04	
	#4	D(−12/−14)	1.4	0.5	0.9	3.3E+05	1.9	0.9	1.1	2.9E+03	8.8E+03	
	#5	D(−12/−14)	−1.5	−2.1	0.6	3.9E+05	0.5	−0.5	1.0	3.0E+03	7.7E+03	
**CV**						**0.27**				**0.09**	**0.32**	

Inter-day experimental variation without and with ESM.

Note: SM8: surgically removed, formalin-fixed, paraffin-embedded (FFPE) sample 8. ISM D (−12/−14) contains *ACTB* IS 10^−12^ M/target gene IS 10^−14^ M that corresponds to *ACTB* IS 600000/target gene IS 6000 molecules. ISM E (−12/−15) contains *ACTB* IS 600000/target gene IS 600 molecules. Cq: quantification cycle. ΔCq: quantification cycle difference of NT and IS: NT Cq - IS Cq. CV: coefficient of variation. NT: native template. IS: internal standard. ISM: internal standards mixture.

### Accuracy

The concentration of each stock (purified) IS was determined using densitometric quantification of the appropriately sized peak after electrophoretic separation on the Agilent Bioanalyzer 2100. Then the appropriate volume of each IS was combined to make an ISM. After preparing the ISM, limiting dilution PCR and Poisson analysis were used to determine the true concentration of each IS in the ISM. Specifically, the stock ISM solution was serially diluted to a concentration expected to contain 40, 20, 10, 7, 4, 2, 1, 0.7, 0.4, 0.1 molecules of each IS in each microliter. The expected frequency of reactions with detectable PCR product at each dilution was tested with real time PCR using the pre-amplification method to increase the signal to background ratio (See the above section: Pre-amplification (1^st^ round PCR) and second-round PCR. As an example, when 1 µl of the dilution expected to contain 0.7 molecules per microliter solution was included in the PCR, the expected frequency of positive reactions was 50.3% by Poisson analysis. The nine replicate samples of each dilution for each gene (*ACTB, MYC, E2F1, CDKN1A*) were measured. For each dilution the observed frequency of positive values (true concentration value) was plotted versus the frequency expected if the concentration determined by Agilent Bioanalyzer 2100 was correct [29].

### Linearity

For each gene, the linearity of the assay was assessed through serial 10-fold dilution of the external standard stock solution (10^−11^ M NT/10^−11^ M IS to 10^−17^ M NT/10^−17^ M IS) or serial dilution keeping IS constant and diluting NT up to 1/80-fold relative to IS and vice versa. For each dilution series, correlation coefficient (r^2^) and slope (linearity) were calculated.

### Imprecision

For each gene, the imprecision was measured as the coefficient of variation (CV) of the copy number measured at each dilution used in the linearity test. The CV was calculated as the standard deviation divided by the mean derived from multiple replicate measurements (at least three). The average CV across all dilutions for each gene, and the average CV across all four genes were calculated.

### Robustness and Interference Tests

The effect of intentionally perturbing PCR conditions was assessed. Conditions altered included PCR volume and concentration of primer, probe, or EDTA [30]. Samples used for this analysis were cDNA reverse transcribed from non-FFPE treated benign lung tissue RNA (Life Technologies, Grand Island, NY) or FFPE-processed, surgically-removed, malignant lung tissue sample 1 or 8 RNA (SM1, SM8).

#### EDTA concentration variation

The effect of variation in PCR EDTA concentration on *MYC* and *ACTB* measurement was assessed in triplicate 20 µl PCR assays containing non-FFPE, benign lung cDNA pre-amplified with ISM. EDTA concentrations tested were 0, 0.5, 1, 1.4, 1.8, 2.2, 2.6, 2.8, 3.0, 3.2, 3.4, 3.6 and 4.0 mM.

### Effect of ESM on Quality-control

The effect of variation in fluorescence intensity on reliability of measurement was tested by varying ratio of [labeled probe]/[unlabeled probe] keeping [total probe] in the PCR constant at 200 nM. PCR assays were conducted for *MYC* and *ACTB* measurement in non-FFPE, benign lung cDNA pre-amplified with ISM. The IS labeled probe concentration in the PCR was kept constant while NT labeled probe was diluted with unlabeled probe to 200, 150, 100, 80, 40, 20, 10, 5, 0 nM or vice versa. Unlabeled probe was obtained from Life Technologies, Grand Island, NY.

Inter-day experimental variation without or with ESM was tested in FFPE sample SM8 cDNA in seven PCR reactions on five different days. The automatically selected Cq values were used to measure *MYC*/10^6^ *ACTB.*

### Statistical Analysis

The transcript abundance value (target gene molecules/10^6^ *ACTB* molecules) for each LCDT gene was measured in triplicate and variation was measured as the CV. We used the Student’s t-test to determine a significant (*P*<0.05) difference in mean LCDT value of the malignant group compared to the benign group. Levene’s test was used to assess the equality of variances in different samples for Student’s t-test using R program (v 2.15.2). The receiver operator characteristic (ROC) plot was generated by GraphPad Prism 6.

## Results

### Analytical Validation of Primers, Probes, and Internal Standard Mixtures

#### Primer efficiency and specificity

Primer efficiency was determined by PCR analysis of serially diluted IS. For each gene, at the dilution predicted to contain a single molecule of IS based on Agilent 2100 concentration analysis, the fraction of measured replicates that had detectable PCR product was consistent with the frequency predicted by Poisson analysis (see Accuracy section below). The Poisson analysis results support the conclusion that the IS concentration was accurate and that the primers had efficiency necessary to generate a detectable signal from a single molecule after 40 cycles [29].

#### Probe specificity

For the first *E2F1* assay design, the NT probe had >10% non-specific binding, so it was re-designed to increase the number of changes in IS compared to NT from four to six base pairs. After the re-design of the *E2F1* IS and its respective probe, non-homologous (non-specific) binding was <1% for both NT and IS probes for all genes, more than meeting our threshold acceptance criteria.

#### Internal standards mixture accuracy

**Figure 2:**
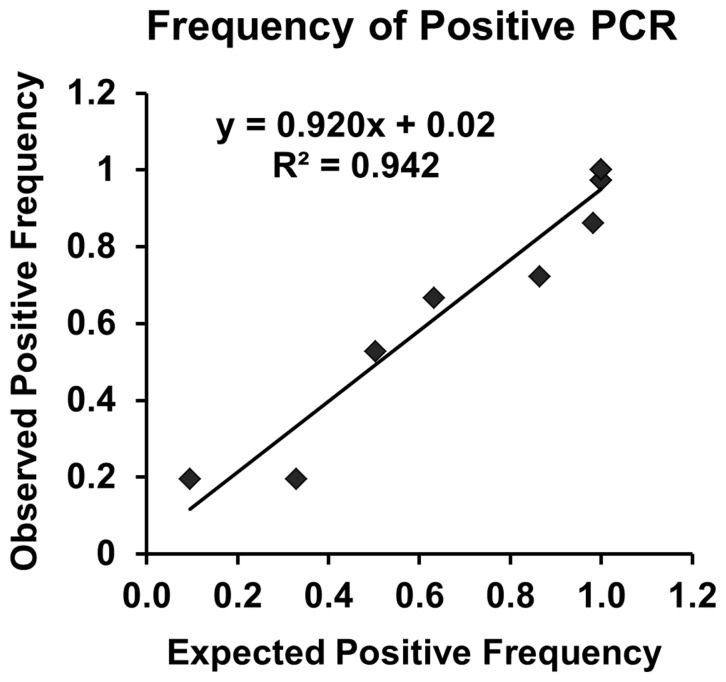
Observed compared to expected positive PCR with limiting dilution. Frequency of observed relative to expected positive PCR signal was measured. Poisson analysis was used to calculate expected positive frequency. Results from the average of nine replicates at each of 10 internal standard mixture dilution points (40, 20, 10, 7, 4, 2, 1, 0.7, 0.4, 0.1 molecules/µl) averaged across the four genes (*ACTB, MYC, E2F1, CDKN1A*) were compiled and plotted. Each gene plot is presented in Figure S2 in File S1.

After the IS were combined into the ISM, the ISM was serially diluted beyond the level expected to contain a single molecule in a PCR assay, and the IS for each of the four genes was PCR-amplified in the PCR assays containing each ISM concentration. The observed frequency of the positive result was highly correlated (R^2^ = 0.94) with the expected positive frequency predicted by Poisson analysis (Figure 2, Figure S2 in File S1), indicating that the intended concentration for each IS in the ISM was accurate.

### Analytical Validation of the Competitive Multiplex Two Color Real-time Method

#### Linearity

**Figure 3:**
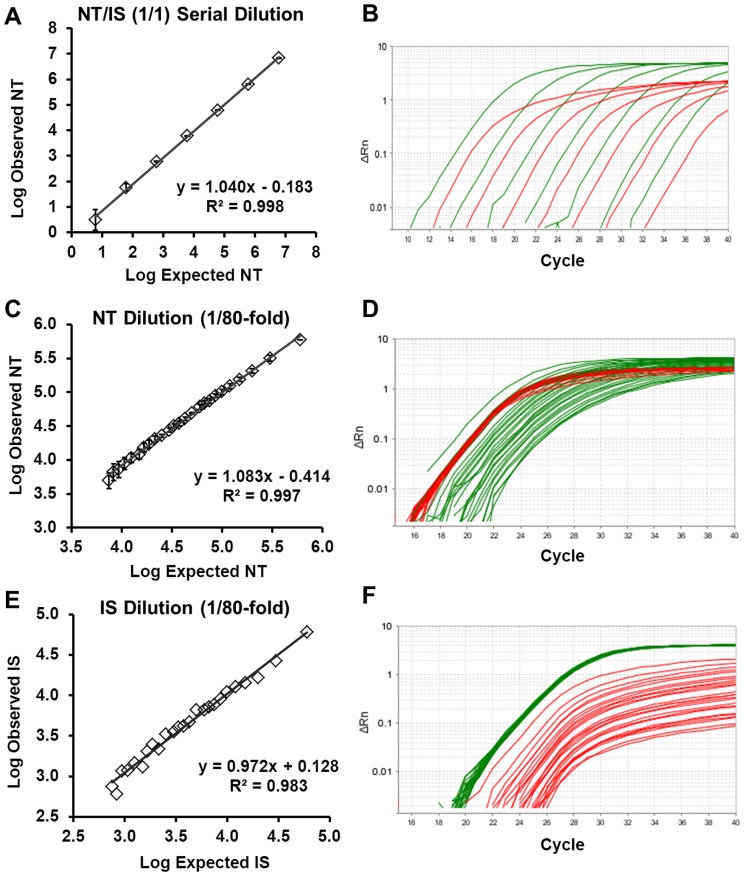
Observed compared to expected E2F1 NT molecule values measured by two-color fluorometric assay in dilution series samples. Linearity graphs (A, C, E) and amplification plots of *E2F1* (B, D, F). (A, B) Serial dilution of external standards mixture (ESM, 1/1 mixture of NT/IS) from 10^−11^ M through 10^−17^ M (triplicate measurements, with error bars). (C, D) NT dilution relative to constant IS from 1/1 NT/IS (10^−12^ M) down to 1/80 (NT/IS) (triplicate measurements with error bars). (E, F) IS dilution relative to constant NT from 1/1 NT/IS (10^−13^ M) down to 1/80-fold (one replicate). NT: native template. IS: internal standard.

The linearity of the two-color fluorometric assay was determined by analysis of serial dilutions of synthetic NT and IS for each gene. In a serial dilution of the stock ESM (a 1/1 mixture of NT/IS) over seven orders of magnitude (from 10^−11^ M through 10^−17^ M), the correlation coefficient for the measurement of each gene relative to its respective IS was >0.99, and the average slope for the signal-to-analyte response was 1.0±0.05 (Figure 3A, B, Figure S3 in File S1).

To more stringently assess linearity, the NT was serially diluted relative to a constant IS concentration of 10^−12^ M and the IS was diluted relative to a constant NT concentration of 10^−13^ M. In the dilutions with NT/IS or IS/NT ratio of <10, the average slope for the four genes (*ACTB, E2F1, MYC, CDKN1A*) was 1.0±0.10 in each set of dilution series. At dilutions with NT/IS or IS/NT >10, there was a slight deviation of the slope from 1.0 (Figure 3C–E, Figure S4 in File S1, S5 in File S1).

#### Imprecision

The imprecision for measurement of the LCDT genes was measured among both the ESM dilution samples and the NT/IS dilution samples.

At each serial 10-fold dilution of ESM (10^−11^ M NT/10^−11^ M IS to 10^−17^ M NT/10^−17^ M IS), the average coefficient of variation (CV) for measurement of each of the four genes was <10% for >60 molecules input (10^−11^ M NT/10^−11^ M IS to 10^−16^ M NT/10^−16^ M IS) and <30% for >6 molecules input (10^−11^ M NT/10^−11^ M IS to 10^−17^ M NT/10^−17^ M IS) with little inter-gene variation (Table S2 in File S1).

Among the NT/IS dilution samples, the average CV among the four LCDT genes was calculated for different ranges of dilution. For an NT dilution from 1/1 to 1/10-fold relative to a constant IS, the average CV among the four genes was 12%. At dilutions beyond 1/10, the CV increased, but from 1/1 to 1/80 NT dilution the average CV was only 20% (Table S3 in File S1). Similar results were observed for an average CV for each of the four genes in the IS dilution relative to a constant NT.

#### Robustness and interference testing

Changing the volumes and/or the concentrations of primers or probes did not lead to significant differences in expression measurement of *MYC* or *ACTB* in FFPE SM1 cDNA with or without pre-amplification (Figure S6 in File S1).

**Figure 4:**
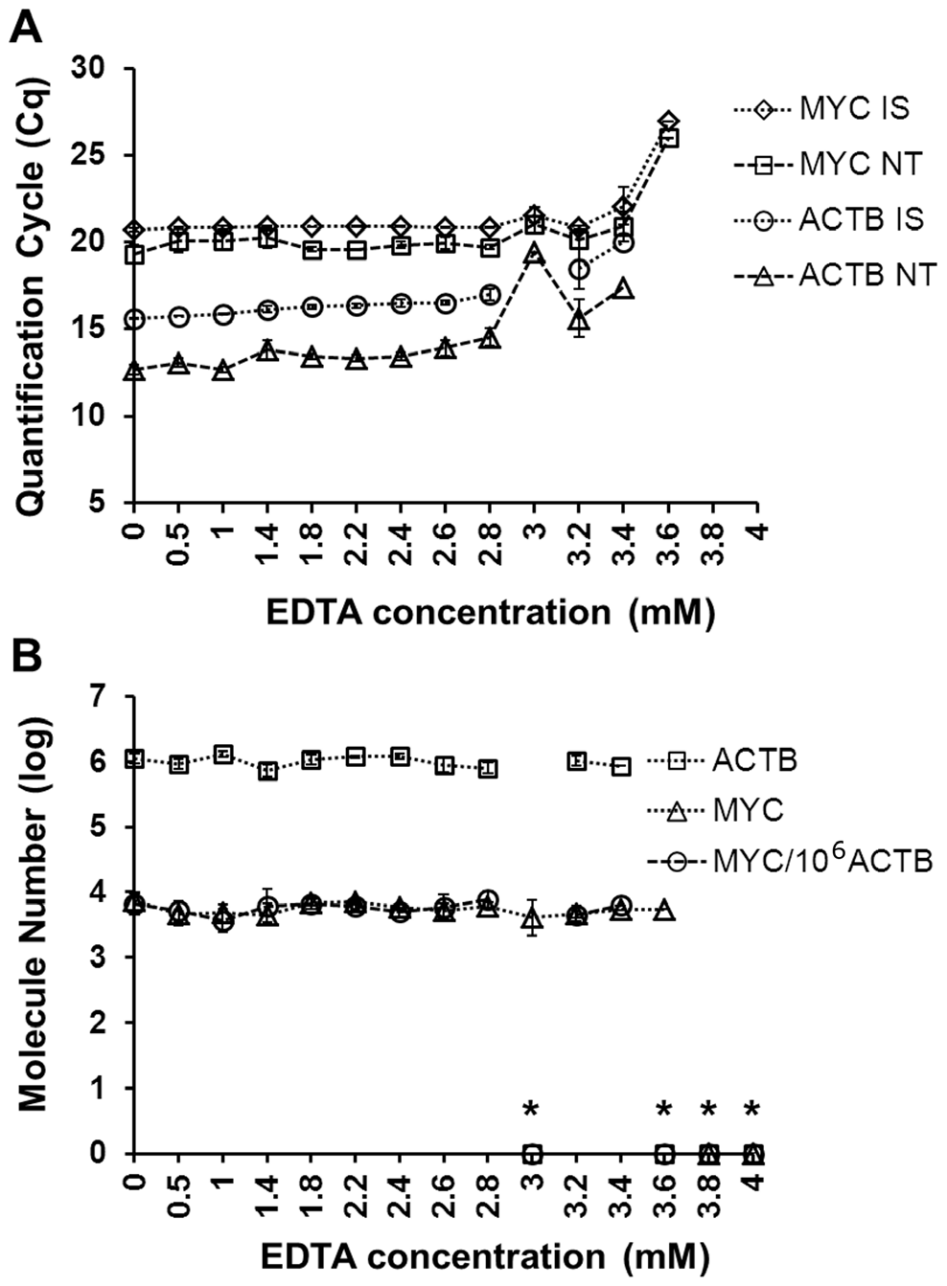
Internal standards control for PCR inhibition by EDTA. *MYC* and *ACTB* were measured in the presence of varying EDTA concentration. (A) Quantification cycle (Cq) values of *MYC* IS, *MYC* NT, *ACTB* IS, *ACTB* NT. (B) Molecules of each gene and normalized value of *MYC*/10^6^ *ACTB* molecules (triplicate measurements) analyzed in benign, non-FFPE lung cDNA reverse transcribed with gene specific primers. NT: native template. IS: internal standard. FFPE: formalin-fixed, paraffin-embedded. The asterisk (*) indicates that Cq values were undetermined by software.

As EDTA concentration was increased, Cq value of each of the four analytes tested in non-FFPE, pre-amplified, benign lung cDNA (*MYC* IS and NT, *ACTB* IS and NT) increased, ultimately resulting in no signal (Figure 4). However, the *MYC* NT and *ACTB* NT values calculated relative to their respective IS were constant, and due to the loss of signal for IS at highest EDTA concentration, no false negative values were reported.

#### Use of ESM to control for variation in fluorescent labeling of probe and selection of threshold

**Figure 5:**
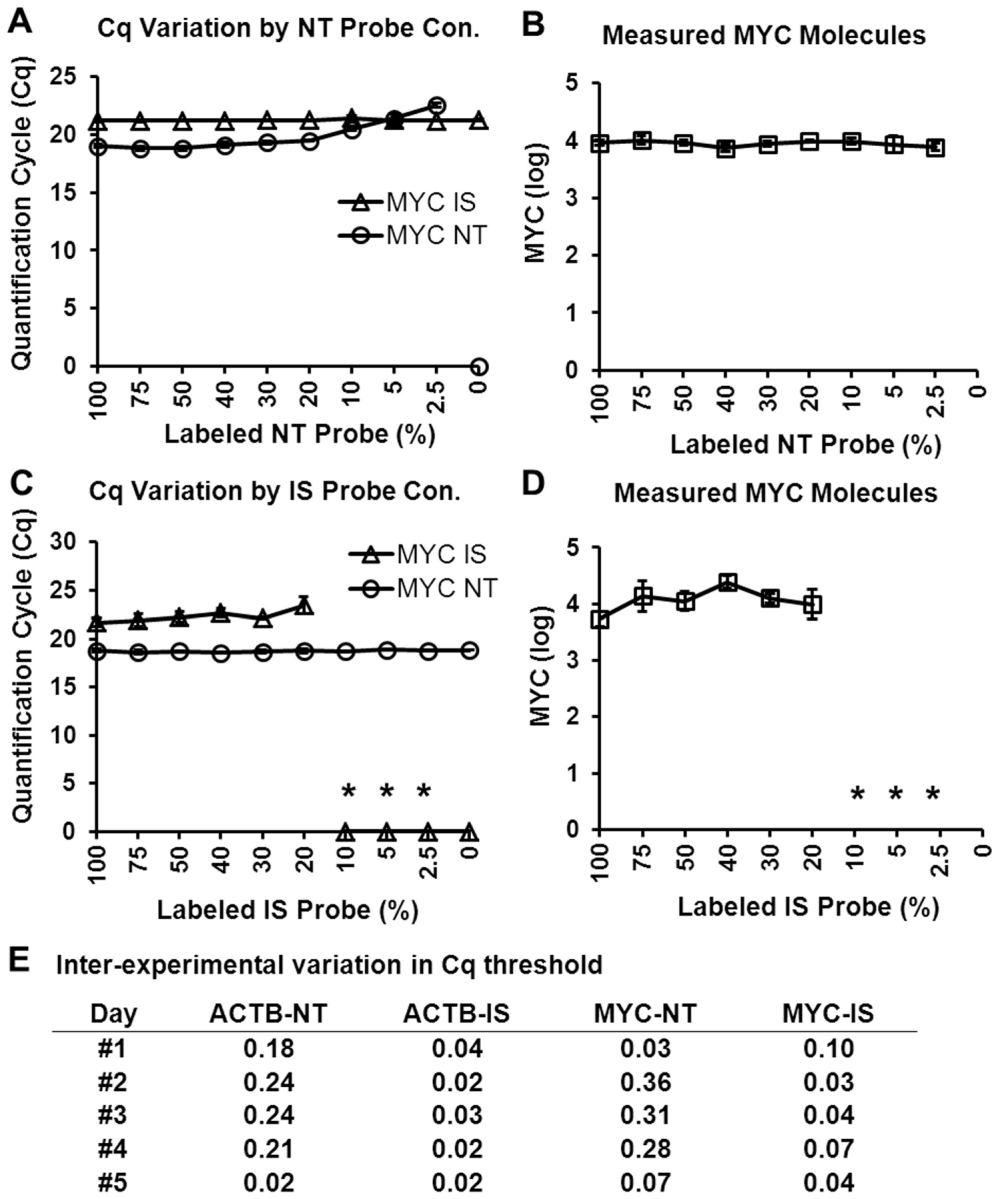
External standards mixture controls for inter-experimental variation in fluor signal or quantification cycle (Cq) selection. (A–D) Effect of diluting labeled probe with unlabeled probe on measurement of *MYC* in benign, non-FFPE lung cDNA reverse transcribed with gene specific primers (triplicate measurements, with error bars). (A, B) NT labeled probe diluted with NT unlabeled probe. (C, D) IS labeled probe diluted with IS unlabeled probe. (E) Effect of inter-day variation in threshold selection on measurement of *MYC* and *ACTB* in surgically removed, FFPE sample 8 (SM8). NT: native template. IS: internal standard. FFPE: formalin-fixed, paraffin-embedded. The asterisk (*) indicates that Cq values were undetermined by software.

The specific activity of probe labeling with fluor (i.e., [labeled probe]/[total probe]) may vary between experiments due to freezing and thawing of probes or due to lot differences, the effect of variation in fluorescence specific activity on measurement of *MY*C in benign, non-FFPE lung cDNA was tested. As the labeled probe concentration decreased in the reaction, the Cq increased (Figure 5A, C). However, this potential source of analytical variation was controlled by correcting the measured lung sample [NT Cq - IS Cq] values relative to the ESM [NT Cq - IS Cq] values (Figure 5B, D). The ESM contained a known 1∶1 concentration of each NT and IS that was constant among experiments, any variation in the observed ESM [NT Cq - IS Cq] relative to the expected value of 0 was attributable to variation in experimental conditions, including fluorescence intensity.

Another potential source of inter-experimental variation is inter-experimental variation in selection of Cq threshold. Even when the Auto Cq mode is used to select automatically the optimal Cq threshold, there was large inter-experimental variation in NT/IS Cq difference based on amplification plot and amount of cDNA loaded (Figure 5E). Thus, whether the threshold was selected through the automatic method or the manual method, there was day-to-day variation in the selected Cq threshold setting. However, because the inter-experimental variation in the Cq threshold had the same effect on sample Cq and ESM Cq, inter-experimental variation in sample Cq was controlled by ESM Cq as described in the previous paragraph. For example, *MYC*/10^6^ *ACTB* was measured in FFPE sample SM8 cDNA in seven PCR replicates on five different days and the Cq threshold value automatically selected in each PCR was different (Figure 5E) resulting in high CV of 0.99. However, with the ESM correction, the CV of measured *MYC*/10^6^ *ACTB* was reduced to 0.32 (Table 2).

### Fitness for Purpose Testing in FFPE Samples

The histomorphologic diagnosis of benign or malignant FFPE samples used in this study is presented in Table S4 in File S1. The RNA yield and purity are presented in Table S5 in File S1.

#### Optimization of FFPE reverse transcription

Efficiency of RT with GSP or RHP was assessed in three (two malignant and one benign) surgical FFPE samples (SM1, SM2, SB1). The average yield of cDNA from 1 µg RNA was more than 50-fold higher with GSP. Based on this, analysis of FFPE samples was conducted with GSP in RT. The RT yield was increased another 4.6-fold by increasing RNA in RT to 5 µg.

#### Effect of pre-amplification

Results for analysis of LCDT genes in sample SM1 with or without pre-amplification were compared to quantify the increase in signal relative to background resulting from pre-amplification and to confirm that it did not significantly alter the result. Importantly, for each gene the signal was increased (Cq decreased) with pre-amplification. Specifically, Cq value decreased for *ACTB*, *MYC*, *E2F1*, and *CDKN1A* by 9, 10, 9 and 10, respectively, following pre-amplification and 1000-fold dilution of the pre-amplification product prior to second round amplification. Yet, because each target was measured relative to a known number of its respective IS molecules, the value measured with the pre-amplification method was not significantly different from that measured with the no pre-amplification (Figure S6 in File S1).

#### Analysis of MYC, E2F1, CDKN1A and ACTB in FFPE samples

**Figure 6:**
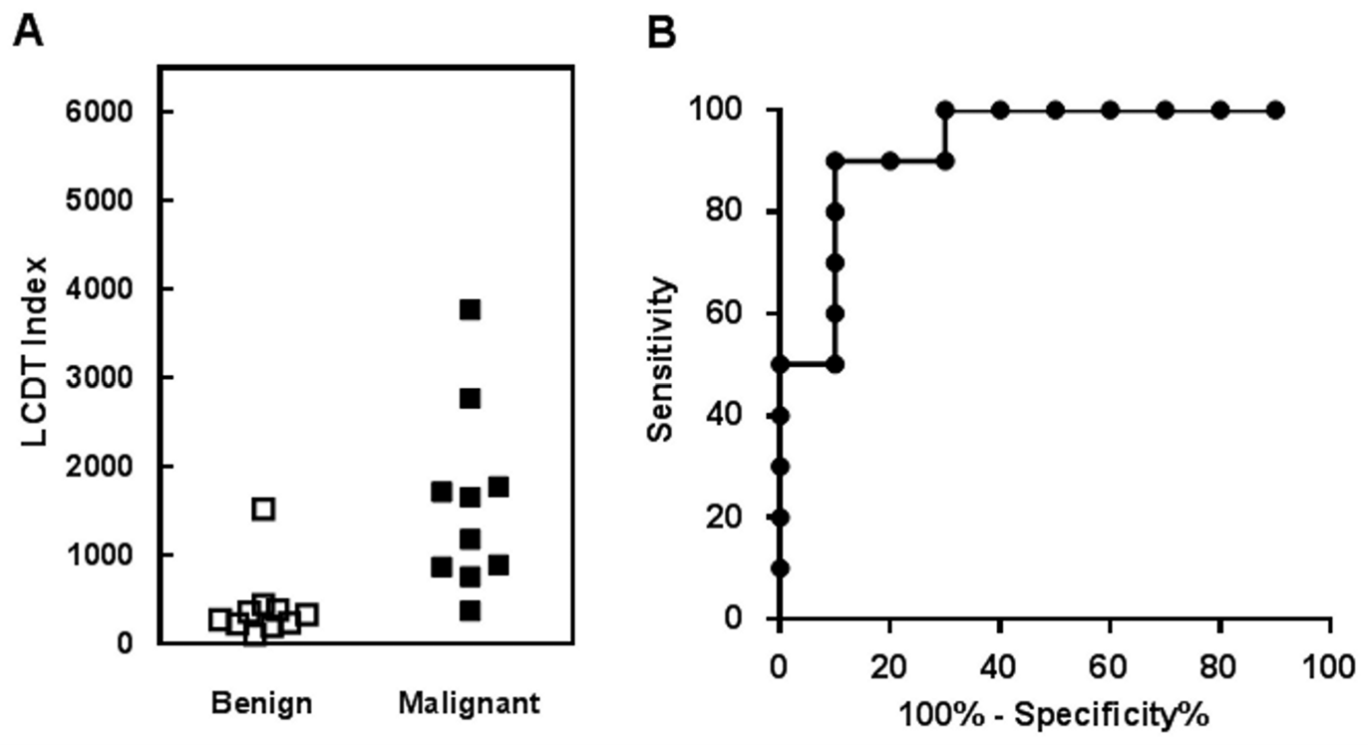
Validation of two-color fluorometric assay in 20 surgically removed, FFPE lung samples. (A) lung cancer diagnostic test (LCDT) index values by diagnostic class. (B) receiver operator characteristic curve (ROC) of LCDT index.

The comparison of the LCDT index in benign and malignant surgical FFPE samples is presented in Figure 6A, and the ROC curve analysis is presented in Figure 6B. Based on the linearity and imprecision results, for analysis of clinical samples, we chose to restrict the conditions for calculation of results to1/10 to 10/1-fold difference between the NT and the IS.

The LCDT optimal cut-off value had 90% specificity and 90% sensitivity to classify samples as cancer or non-cancer, similar to previous reports with non-FFPE fine needle aspirate (FNA) samples [31], [32]. The ROC area under the curve was 0.93 with a 95% confidence interval of 0.82 to 1.04 and the *P*-value of Student’s t-test for stratification of malignant from non-malignant was 0.0061. The average CV among surgical FFPE samples for measurement of *MYC, E2F1*, and *CDKN1A* relative to *ACTB* was 0.27, 0.41, and 0.26, respectively (Table 3). These data confirm fitness for purpose of this optimized LCDT test in FFPE samples.

**Table 3:** Each gene measurement and lung cancer diagnostic test (LCDT) index in surgically removed, formalin-fixed, paraffin-embedded lung samples (n = 20).

	*MYC*			*E2F1*			*CDKN1A*			LCDT
Benign	AVE	SD	CV	AVE	SD	CV	AVE	SD	CV	
SB1	1.5E+4	1.2E+4	0.83	6.7E+2	3.9E+2	0.58	3.6E+4	1.5E+4	0.41	2.8E+2
SB2	2.1E+4	8.6E+3	0.42	1.3E+3	9.6E+2	0.77	5.8E+4	9.5E+3	0.16	4.5E+2
SB3	4.1E+3	4.9E+2	0.12	2.8E+3	1.0E+3	0.35	3.1E+4	2.2E+3	0.07	3.8E+2
SB4	7.4E+3	1.0E+3	0.14	1.5E+3	4.5E+2	0.30	3.0E+4	2.6E+3	0.09	3.6E+2
SB5	1.8E+3	3.1E+2	0.17	1.4E+3	1.7E+2	0.12	1.1E+4	5.7E+2	0.05	2.4E+2
SB6	3.2E+3	5.7E+2	0.18	1.3E+3	2.7E+2	0.20	4.1E+4	1.0E+4	0.25	1.1E+2
SB7	8.1E+3	2.4E+3	0.30	7.5E+3	3.7E+3	0.49	4.0E+4	1.3E+4	0.32	1.5E+3
SB8	9.5E+3	3.1E+3	0.33	1.3E+3	8.5E+2	0.65	5.4E+4	3.2E+3	0.06	2.3E+2
SB9	6.6E+3	1.2E+3	0.18	7.1E+2	4.8E+2	0.69	2.4E+4	7.4E+3	0.31	2.0E+2
SB10	2.7E+4	6.8E+3	0.26	9.5E+2	6.3E+2	0.66	7.6E+4	2.6E+4	0.35	3.3E+2
**AVE**			**0.29**			**0.48**			**0.21**	
**Malignant**	**AVE**	**SD**	**CV**	**AVE**	**SD**	**CV**	**AVE**	**SD**	**CV**	
SM1	1.5E+4	3.8E+3	0.25	6.1E+3	2.7E+3	0.45	5.1E+4	2.3E+4	0.45	1.8E+3
SM2	7.4E+3	1.6E+3	0.22	8.2E+3	2.3E+3	0.28	2.2E+4	5.5E+3	0.25	2.8E+3
SM3	1.2E+4	4.0E+3	0.33	1.2E+3	5.0E+2	0.43	1.6E+4	4.4E+3	0.28	8.9E+2
SM4	1.4E+4	2.7E+3	0.20	1.2E+3	5.2E+2	0.43	4.4E+4	7.7E+3	0.17	3.8E+2
SM5	1.2E+4	3.1E+3	0.26	1.8E+3	3.5E+2	0.20	1.8E+4	5.1E+3	0.29	1.2E+3
SM6	7.7E+3	1.8E+3	0.23	4.2E+3	1.9E+3	0.45	1.9E+4	3.7E+3	0.20	1.7E+3
SM7	9.3E+3	2.9E+3	0.31	2.0E+4	6.4E+3	0.31	5.0E+4	1.7E+4	0.33	3.8E+3
SM8	9.8E+3	3.4E+3	0.35	2.8E+3	1.3E+3	0.47	3.6E+4	1.4E+4	0.39	7.6E+2
SM9	1.3E+4	1.6E+3	0.12	3.2E+3	1.3E+2	0.04	2.6E+4	8.5E+3	0.33	1.7E+3
SM10	1.0E+4	1.9E+3	0.19	2.4E+3	9.9E+2	0.41	2.9E+4	1.1E+4	0.40	8.7E+2
**AVE**			**0.25**			**0.35**			**0.31**	

Note: SB: surgically removed, benign sample. SM: surgically removed, malignant sample. AVE: average. SD: standard deviation. CV: coefficient of variation.

## Discussion

Here we report the analytical validation and fitness for purpose testing of an RT-qPCR method suitable for reliable analysis of FFPE samples. Key features of this optimized method are highlighted here.

### Internal Standards Provide Quality Control

The endogenous amount (NT) of each of multiple genes was measured relative to a known number of respective IS molecules. Each IS amplified with the same efficiency as the NT, and this controlled for inter-sample variation in PCR interfering substances and inter-experimental variation in PCR reagent quality or quantity or thermal cycling conditions as previously described [30], [33], [34]. Key to the elimination of inter-experimental variation when measuring multiple genes was use of the same ISM comprising a known concentration of IS for each of the genes to be measured [16]. The ISM was both stable and simple and inexpensive to prepare.

### Two-color Fluorometric Real-time PCR

For reliable analysis of FFPE samples, it is important to use primers that yield short PCR products (e.g. 60−80 base pairs). Such products are readily quantified using real-time PCR. Competitive PCR analysis involves simultaneous quantification of each target gene NT and its respective IS. To do this by real-time PCR requires inclusion of two different sequence-specific probes in the same PCR reaction, each with a different fluor [12], [13], [35]. Calculating each NT analyte relative to its respective IS requires an additional measurement, and, in some studies, this may be associated with a tendency to increased imprecision [36]. However, as previously reported, the imprecision observed in this study was less than 10% except for the measurement of very low copy numbers (<60 copies), at which point imprecision is determined largely by the natural law governing stochastic sampling variation rather than method-specific characteristics [16].

### ESM Controlled for Inter-run Variation in Probe Fluorescence

Multiple different factors may cause inter-experimental variation in fluor signal detection including variation in fluor concentration (Figure 5A–D), variation in cycle threshold setting (Figure 5E), and as yet unknown sources. The use of the ESM significantly reduced these sources of inter-experimental variation (Table 2). In addition to use in multiple analyte assays, such as the one presented here, this approach is applicable to single analyte two-color fluorometric assays [12], [13] and may demonstrate similar utility if so employed.

### Multiplex Pre-amplification was Enabled by Use of Internal Standards

Using IS in conjunction with multiplex pre-amplification enabled reliable analysis of even lowly expressed genes in very small amounts of cDNA. Specifically, it was possible to determine the starting number of NT molecules, even after two rounds of PCR, by measuring the NT signal relative to the IS signal (Figure S6 in File S1). This is because a known number of its respective IS molecules was included in the pre-amplification reaction for each gene, and because the NT and IS amplified with the same efficiency [20].

Competitive multiplex pre-amplification improved measurement of FFPE samples in the following ways. First, cDNA consumption was reduced. In the multiplex pre-amplification, reduction in cDNA consumption depends on the number of targets and reference genes. Thus, in this study involving only three target genes and a single reference gene, cDNA consumption was reduced four-fold. We have previously conducted competitive multiplex pre-amplification with 96 genes, and this enabled a marked reduction in cDNA required for measurement of each gene [20], [37].

Second, pre-amplification markedly increased signal above the background signal typically observed in the no template control at 35 Cq. Specifically, with one round of PCR (no pre-amplification) the Cq for each NT and IS ranged from 20–35. In contrast, using pre-amplified and 1000-fold diluted samples, the Cq for each NT and IS after a second round of PCR ranged from 11–26. The amount of dilution of first round amplification product can be reduced if necessary to ensure sufficient signal in the second round for very low input of sample cDNA. Further, for analysis of FNA FFPE samples with very low input cDNA, the higher signal following pre-amplification is associated with better precision than no pre-amplification (data not shown).

### Gene Specific Reverse Transcription

We recently reported that use of gene specific priming in RT increases yield of cDNA by 10–100 fold compared to oligo dT or random hexamer priming when applied to RNA from human peripheral blood leukocytes [26]. Because FFPE treatment typically reduces yield of cDNA from RNA by 100-fold, we evaluated the utility of gene specific priming relative to random hexamer priming to increase signal. The more than 50-fold increase in cDNA yield with gene specific priming RT compared to random hexamer priming RT observed in this study is consistent with our results from the prior study with leukocytes.

### Fitness of Method for FFPE Sample Analysis

Fitness of this two color fluorometric method for analysis of FFPE samples was evaluated by measuring a previously described test for lung cancer diagnosis for non-FFPE FNA samples [31], [32] in a small number of surgical FFPE benign and malignant lung samples. The results support the utility of this optimized method for analysis of FFPE samples. Specifically, imprecision was acceptable, and the optimal cut-off for the LCDT had similar accuracy in separating benign from malignant compared to what was reported previously for fresh FNA samples [31], [32]. These results support the conclusion that the method presented here is suitable for use in a planned clinical validation trial in which the LCDT will be evaluated for utility to augment cytomorphology in analysis of FNA cell block FFPE samples.

### Summary

Successful analytical validation described here of the quality-controlled two-color fluorometric real-time PCR method for analysis of the LCDT in FFPE samples supports use of this approach in development and implementation of promising RT-qPCR based diagnostic tests that require analysis of RNA extracted from FFPE samples.

## Supporting Information

File S1 **Supporting information.** Table S1, (A) Composition of Internal Standards Mixture (ISM) A–F. (B) Steps to calculate *MYC*/10^6^ *ACTB* value for surgically removed, malignant sample 1 (SM1). To quantify the copy number for each target gene native template (NT) in a cDNA sample, 1) the ΔCq: [NT Cq - IS Cq]_Sample_ for unknown sample and the average of two concentrations of ESM ΔCq: [NT Cq - IS Cq]_ESM_ were calculated, 2) The corrected delta Cq was calculated as: [NT Cq - IS Cq]_Sample_ - [NT Cq - IS Cq]_ESM_, 3) 2^(− corrected delta Cq)^ was multiplied times the known number of input IS copies in the reaction to obtain the gene NT copy number, and 4) each target gene NT value was normalized to the *ACTB* loading control gene NT value, and presented as target gene NT molecules/10^6^ *ACTB* molecules. ISM D (−13/−15) contains *ACTB* IS 10^−13^ M/each target gene IS 10^−15^ M that corresponds to *ACT*B IS 60000/each target gene IS 600 molecules. Figure S1, Schematic plot of experiment set up for 96 well plate. After dilution of pre-amplified PCR product containing cDNA and internal standards mixture (ISM), an aliquot of each diluted products was distributed into individual wells for 2^nd^ round amplification for each individual gene native template (NT) and respective internal standard (IS) using gene-specific primers and probes. ISM C(−13/−15) was presented in the figure as an example, containing *ACTB* IS 10^−13^ M/each target gene IS 10^−15^ M corresponding to *ACTB* IS 60000 molecules/each target gene IS 600 molecules. The PCR amplification plots for *ACTB* from the two external standard mixtures (ESM), NT and IS each at 10^−13^ M or NT and IS each at 10^−14^ M, are presented in one plot in the middle. Green is NT and red is IS in the plot. SM: surgically removed, malignant sample. NTC: no template control. Figure S2, Observed compared to expected positive PCR with limiting dilution PCR for each gene. Pre-amplification method was used for testing 9 replicates. Each of 10 dilution points of internal standards mixture (ISM) (40, 20, 10, 7, 4, 2, 1, 0.7, 0.4, 0.1 molecules) was multiplex pre-amplified nine times then observed PCR positivity for each gene at 2^nd^ amplification. Figure S3, Observed compared to expected native template (NT) molecule values measured by two-color fluorometric assay in external standards mixture (ESM) dilution series samples. ESM (1/1 mixture of NT and internal standard (IS)) was serial 10-fold diluted from NT 10^−11^ M/IS 10^−11^ M to NT 10^−17^ M/IS 10^−17^ M and each dilution sample analyzed in triplicate. Figure S4, Observed compared to expected native template (NT) molecule values measured by two-color fluorometric assay in serially diluted synthetic NT relative to constant synthetic internal standard (IS) dilution series samples. *ACTB, MYC, E2F1, or CDKN1A* synthetic NT was serially diluted relative to constant synthetic IS, starting with 1/1 NT/IS mixture at 10^−12^ M. A, C, E, G: Linearity from 1/1 to 1/10-fold NT dilution. B, D, F, H: Linearity from 1/1 to 1/80-fold NT dilution. We assessed 1/1, 1/2, 1/3, 1/4, 1/5, 1/6, 1/7, 1/8, 1/9, 1/10, 1/12, 1/14, 1/16, 1/18, 1/20, 1/24, 1/28, 1/32, 1/36, 1/40, 1/48, 1/56, 1/64, 1/72, 1/80-fold dilutions of NT relative to constant IS. Data were analyzed with triplicate measurement. Figure S5, Observed compared to expected native template (NT) molecule values measured by two-color fluorometric assay in serially diluted synthetic internal standard (IS) relative to constant synthetic NT dilution series samples. *ACTB, MYC, E2F1, or CDKN1A* synthetic IS was serially diluted relative to constant synthetic NT starting with 1/1 of NT/IS mixture at 10^−13^ M. A, C, E, G: Linearity from 1/1 to 1/10-fold IS dilution. B, D, F, H: Linearity from 1/1 to 1/80-fold IS dilution. We assessed 1/1, 1/2, 1/3, 1/4, 1/5, 1/6, 1/7, 1/8, 1/9, 1/10, 1/12, 1/14, 1/16, 1/18, 1/20, 1/24, 1/28, 1/32, 1/36, 1/40, 1/48, 1/56, 1/64, 1/72, 1/80-fold dilution of IS relative to constant NT (one replicate measurement). Auto Cq could not be generated for the 1/56, 1/64, 1/72, 1/80-fold IS dilutions of *ACTB*. Table S2, Effect of external standards mixture (ESM) dilution on precision in measurement of each lung cancer diagnostic test (LCDT) genes by two-color fluorometric real-time assay. A serially diluted 1∶1 ratio of native template (NT): internal standard (IS) from 10^−11^ M through 10^−17^ M was analyzed in triplicate. Table S3, Effect of native template (NT) dilution relative to internal standard (IS) on precision in measurement of the lung cancer diagnostic test (LCDT) genes by two-color fluorometric real-time assay. For each gene serial dilution of synthetic NT up to 1/80-fold relative to constant IS was measured at each dilution. At each NT dilution points the compiled data across the four genes (*ACTB, MYC, E2F1, CDKN1A*) in triplicate measurements were presented. Figure S6, Effect of PCR reaction conditions on lung cancer diagnostic test (LCDT) gene expression values measured in cDNA with or without pre-amplification. A: pre-amplification. B: no pre-amplification. Surgically removed, formalin-fixed, paraffin-embedded, malignant sample 1(SM1) reverse transcribed with gene specific primers was used. The reference optimal PCR condition was 20 µl reaction volume (2V), 800 nM of primers (1Pm) and 200 nM of probes (1Pb). To test robustness, we reduced volume by half, and/or doubled primer or probe concentration in each of the two conditions (pre-amp or no pre-amp). Table S4, Histomorphological diagnosis of surgically removed, formalin-fixed, paraffin-embedded samples. Table S5, Total RNA sample quantity and purity assessment (A) (n = 20).
(DOCX)

## References

[1] Overdevest JB, Theodorescu D, Lee JK (2009) Utilizing the Molecular Gateway: The Path to Personalized Cancer Management. Clinical Chemistry 55: 684–697. 19246616 10.1373/clinchem.2008.118554 PMC4529063

[2] Poste G, Carbone DP, Parkinson DR, Verweij J, Hewitt SM, et al (2012) Leveling the Playing Field: Bringing Development of Biomarkers and Molecular Diagnostics up to the Standards for Drug Development. Clinical Cancer Research 18: 1515–1523. 22422403 10.1158/1078-0432.CCR-11-2206 PMC3307147

[3] Coyle VM, Johnston PG (2010) Genomic markers for decision making: what is preventing us from using markers? Nature Reviews Clinical Oncology 7: 90–97. 10.1038/nrclinonc.2009.214 20010899

[4] Khleif SN, Doroshow JH, Hait WN, Colla A-F-NCB (2010) AACR-FDA-NCI Cancer Biomarkers Collaborative Consensus Report: Advancing the Use of Biomarkers in Cancer Drug Development. Clinical Cancer Research 16: 3299–3318. 20501613 10.1158/1078-0432.CCR-10-0880

[5] Antonov J, Goldstein DR, Oberli A, Baltzer A, Pirotta M, et al (2005) Reliable gene expression measurements from degraded RNA by quantitative real-time PCR depend on short amplicons and a proper normalization. Laboratory Investigation 85: 1040–1050. 15951835 10.1038/labinvest.3700303

[6] Chung JY, Braunschweig T, Hewitt SM (2006) Optimization of recovery of RNA from formalin-fixed, paraffin-embedded tissue. Diagn Mol Pathol 15: 229–236. 17122651 10.1097/01.pdm.0000213468.91139.2d

[7] Fleige S, Pfaffl MW (2006) RNA integrity and the effect on the real-time qRT-PCR performance. Mol Aspects Med 27: 126–139. 16469371 10.1016/j.mam.2005.12.003

[8] Godfrey TE, Kim SH, Chavira M, Ruff DW, Warren RS, et al (2000) Quantitative mRNA expression analysis from formalin-fixed, paraffin-embedded tissues using 5′ nuclease quantitative reverse transcription-polymerase chain reaction. J Mol Diagn 2: 84–91. 11272893 10.1016/S1525-1578(10)60621-6 PMC1906896

[9] Sen K, Fout GS, Haugland R, Moulton C, Grimm A, et al. (2004) Quality assurance/Quality control Guidance for Laboratories Performing PCR Analyses on Environmental Samples. United States Evironmental Protection Agency: EPA 815-B-04–001. Available:http://www.epa.gov/ogwdw/ucmr/ucmr1/pdfs/guidance_ucmr1_qa-qc.pdf. Accessed. September 2013.

[10] Anonymous (2005) Microbiology of food and animal feeding stuffs-Polymerase chain reaction (PCR) for the detection of food-borne pathogens-General requirements and definitions. International Organization for Standardization (ISO): BS EN ISO 22174. https://www.iso.org/obp/ui/#iso:std:iso:22174:ed-1:v1:en. Accessed September 2013.

[11] Sen K, Fout GS, Haugland R, Moulton C, Grimm A, et al. (2010) Draft Guidance for Industry and Food and Drug Administration staff; Establishing the Performance Characteristics of *in vitro* Diagnostic Devices for the Detection of *Clostridium difficile* Health and Human Services, Food and Drug Administration (FDA). Available: http://www.fda.gov/MedicalDevices/DeviceRegulationandGuidance/GuidanceDocuments/ucm234868.htm. Accessed Sep 2013.

[12] Schumacher W, Frick E, Kauselmann M, Maier-Hoyle V, van der Vliet R, et al (2007) Fully automated quantification of human immunodeficiency virus (HIV) type 1 RNA in human plasma by the COBAS (R) AmpliPrep/COBAS (R) TaqMan (R) system. Journal of Clinical Virology 38: 304–312. 17329164 10.1016/j.jcv.2006.12.022

[13] Gelderblom HC, Menting S, Beld MG (2006) Clinical performance of the new Roche COBAS (R) TaqMan HCV test and high pure system for extraction, detection and quantitation of HCV RNA in plasma and serum. Antiviral Therapy 11: 95–103. 16518965

[14] Swanson P, Huang SH, Holzmayer V, Bodelle P, Yamaguchi J, et al (2006) Performance of the automated Abbott RealTime (TM) HIV-1 assay on a genetically diverse panel of specimens from Brazil. Journal of Virological Methods 134: 237–243. 16510195 10.1016/j.jviromet.2006.01.012

[15] Apostolakos MJ, Schuermann WH, Frampton MW, Utell MJ, Willey JC (1993) Measurement of gene expression by multiplex competitive polymerase chain reaction. Anal Biochem 213: 277–284. 8238902 10.1006/abio.1993.1421

[16] Canales RD, Luo Y, Willey JC, Austermiller B, Barbacioru CC, et al (2006) Evaluation of DNA microarray results with quantitative gene expression platforms. Nat Biotechnol 24: 1115–1122. 16964225 10.1038/nbt1236

[17] Willey JC, Crawford EL, Knight CR, Warner KA, Motten CA, et al (2004) Standardized RT-PCR and the standardized expression measurement center. Methods Mol Biol 258: 13–41. 14970455 10.1385/1-59259-751-3:13

[18] Wang AM, Doyle MV, Mark DF (1989) Quantitation of Messenger-Rna by the Polymerase Chain-Reaction. Proceedings of the National Academy of Sciences of the United States of America 86: 9717–9721. 2481313 10.1073/pnas.86.24.9717 PMC298572

[19] Gilliland G, Perrin S, Blanchard K, Bunn HF (1990) Analysis of Cytokine Messenger-Rna and Dna - Detection and Quantitation by Competitive Polymerase Chain-Reaction. Proceedings of the National Academy of Sciences of the United States of America 87: 2725–2729. 2181447 10.1073/pnas.87.7.2725 PMC53763

[20] Crawford EL, Warner KA, Khuder SA, Zahorchak RJ, Willey JC (2002) Multiplex standardized RT-PCR for expression analysis of many genes in small samples. Biochem Biophys Res Commun 293: 509–516. 12054630 10.1016/S0006-291X(02)00243-7

[21] Farragher SM, Tanney A, Kennedy RD, Paul Harkin D (2008) RNA expression analysis from formalin fixed paraffin embedded tissues. Histochem Cell Biol 130: 435–445. 18679706 10.1007/s00418-008-0479-7

[22] Masuda N, Ohnishi T, Kawamoto S, Monden M, Okubo K (1999) Analysis of chemical modification of RNA from formalin-fixed samples and optimization of molecular biology applications for such samples. Nucleic Acids Res 27: 4436–4443. 10536153 10.1093/nar/27.22.4436 PMC148727

[23] Cronin M, Pho M, Dutta D, Stephans JC, Shak S, et al (2004) Measurement of gene expression in archival paraffin-embedded tissues: development and performance of a 92-gene reverse transcriptase-polymerase chain reaction assay. Am J Pathol 164: 35–42. 14695316 10.1016/S0002-9440(10)63093-3 PMC1602211

[24] DeMuth JP, Jackson CM, Weaver DA, Crawford EL, Durzinsky DS, et al (1998) The gene expression index c-myc x E2F-1/p21 is highly predictive of malignant phenotype in human bronchial epithelial cells. Am J Respir Cell Mol Biol 19: 18–24. 9651176 10.1165/ajrcmb.19.1.3077

[25] Ellison SLR, Williams AS, (Eds) (2012) Eurachem/CITAC guide: Quantifying Uncertainty in Analytical Measurement. Available: www.eurachem.org. Accessed September 2013.

[26] Stanoszek LM, Crawford EL, Blomquist TM, Warns JA, Willey PFS, et al (2013) Quality Control Methods for Optimal BCR-ABL1 Clinical Testing in Human Whole Blood Samples. Journal of Molecular Diagnostics 15: 391–400. 23541592 10.1016/j.jmoldx.2013.02.004 PMC3644732

[27] Heid CA, Stevens J, Livak KJ, Williams PM (1996) Real time quantitative PCR. Genome Research 6: 986–994. 8908518 10.1101/gr.6.10.986

[28] Bustin SA, Benes V, Garson JA, Hellemans J, Huggett J, et al (2009) The MIQE Guidelines: Minimum Information for Publication of Quantitative Real-Time PCR Experiments. Clinical Chemistry 55: 611–622. 19246619 10.1373/clinchem.2008.112797

[29] Sykes PJ, Neoh SH, Brisco MJ, Hughes E, Condon J, et al (1992) Quantitation of targets for PCR by use of limiting dilution. Biotechniques 13: 444–449. 1389177

[30] Huggett JF, Novak T, Garson JA, Green C, Morris-Jones SD, et al (2008) Differential susceptibility of PCR reactions to inhibitors: an important and unrecognised phenomenon. BMC research notes 1: 70. 18755023 10.1186/1756-0500-1-70 PMC2564953

[31] Warner KA, Crawford EL, Zaher A, Coombs RJ, Elsamaloty H, et al (2003) The c-myc x E2F-1/p21 interactive gene expression index augments cytomorphologic diagnosis of lung cancer in fine-needle aspirate specimens. J Mol Diagn 5: 176–183. 12876208 10.1016/S1525-1578(10)60470-9 PMC1907329

[32] Graves TG, Harr MW, Crawford EL, Willey JC (2006) Stable low-level expression of p21(WAF1/CIP1) in A549 human bronchogenic carcinoma cell line-derived clones down-regulates E2F1 mRNA and restores cell proliferation control. Mol Cancer 5. 10.1186/1476-4598-5-1 PMC1368995 16403226

[33] Sidor IF, Dunn JL, Tsongalis GJ, Carlson J, Frasca S Jr (2013) A multiplex real-time polymerase chain reaction assay with two internal controls for the detection of Brucella species in tissues, blood, and feces from marine mammals. Journal of veterinary diagnostic investigation : official publication of the American Association of Veterinary Laboratory Diagnosticians, Inc 25: 72–81. 10.1177/1040638712470945 23345271

[34] Tichopad A, Kitchen R, Riedmaier I, Becker C, Stahlberg A, et al (2009) Design and Optimization of Reverse-Transcription Quantitative PCR Experiments. Clinical Chemistry 55: 1816–1823. 19643838 10.1373/clinchem.2009.126201

[35] Caliendo AM, Valsamakis A, Bremer JW, Ferreira-Gonzalez A, Granger S, et al (2011) Multilaboratory Evaluation of Real-Time PCR Tests for Hepatitis B Virus DNA Quantification. Journal of Clinical Microbiology 49: 2854–2858. 21697326 10.1128/JCM.00471-11 PMC3147738

[36] Stenman J, Orpana A (2001) Accuracy in amplification. Nature Biotechnology 19: 1011–1012. 10.1038/nbt1101-1011b 11689839

[37] Blomquist TM, Crawford EL, Lovett JL, Yeo J, Stanoszek LM, et al.. (2013) Targeted RNA-sequencing with competitive multiplex-PCR amplicon libraries. PLoS One: “In press”. 10.1371/journal.pone.0079120 PMC3827295 24236095

