# Supplementary material for: A Multiplex Two-Color Real-Time PCR Method for Quality-Controlled Molecular Diagnostic Testing of FFPE Samples
Source: PLoS One. 2014 Feb 21;9(2):e89395. doi: 10.1371/journal.pone.0089395 (PMC3931751; doi:10.1371/journal.pone.0089395)
Supplement: File S1 — Supporting information. Table S1, (A) Composition of Internal Standards Mixture (ISM) A–F. (B) Steps to calculate MYC/106 ACTB value for surgically removed, malignant sample 1 (SM1). To quantify the copy number for each target gene native template (NT) in a cDNA sample, 1) the ΔCq: [NT Cq - IS Cq]Sample for unknown sample and the average of two concentrations of ESM ΔCq: [NT Cq - IS Cq]ESM were calculated, 2) The corrected delta Cq was calculated as: [NT Cq - IS Cq]Sample - [NT Cq - IS Cq]ESM, 3) 2(− corrected delta Cq) was multiplied times the known number of input IS copies in the reaction to obtain the gene NT copy number, and 4) each target gene NT value was normalized to the ACTB loading control gene NT value, and presented as target gene NT molecules/106 ACTB molecules. ISM D (−13/−15) contains ACTB IS 10−13 M/each target gene IS 10−15 M that corresponds to ACTB IS 60000/each target gene IS 600 molecules. Figure S1, Schematic plot of experiment set up for 96 well plate. After dilution of pre-amplified PCR product containing cDNA and internal standards mixture (ISM), an aliquot of each diluted products was distributed into individual wells for 2nd round amplification for each individual gene native template (NT) and respective internal standard (IS) using gene-specific primers and probes. ISM C(−13/−15) was presented in the figure as an example, containing ACTB IS 10−13 M/each target gene IS 10−15 M corresponding to ACTB IS 60000 molecules/each target gene IS 600 molecules. The PCR amplification plots for ACTB from the two external standard mixtures (ESM), NT and IS each at 10−13 M or NT and IS each at 10−14 M, are presented in one plot in the middle. Green is NT and red is IS in the plot. SM: surgically removed, malignant sample. NTC: no template control. Figure S2, Observed compared to expected positive PCR with limiting dilution PCR for each gene. Pre-amplification method was used for testing 9 replicates. Each of 10 dilution points of internal standards mix [file pone.0089395.s001.docx]

**Supporting Information**

**Table S1**.

**(A) ISM composition**

| **ISM (M)** | ***ACTB* IS mol./µl** | **Target Gene IS mol./µl** |
| --- | --- | --- |
| A(-12/-11) | 600000 | 6000000 |
| B(-12/-12) | 600000 | 600000 |
| C(-12/-13) | 600000 | 60000 |
| D(-12/-14) | 600000 | 6000 |
| E(-12/-15) | 600000 | 600 |
| F(-12/-16) | 600000 | 60 |

**(B) Example of *MYC*/10^6^ *ACTB* calculation in Sample SM1**

| Samples | ISM (M) | NT Cq | IS Cq | ΔCq | Ave. of ESM ΔCq | Corrected ΔCq | 2^(-corrected ΔCq)^ | IS  mol. | Target  mol. | *MYC*/  10^6^ *ACTB* |
| --- | --- | --- | --- | --- | --- | --- | --- | --- | --- | --- |
| ESM 10^-13^M |  | 13.9 | 16.8 |  | -2.8 |  |  |  |  |  |
| ESM 10^-14^M |  | 17.6 | 20.3 |  |  |  |  |  | ***ACTB*** |  |
| **SM1** | D(-13/-15) | 14.0 | 17.7 | -3.7 | (+2.8) | -0.9 | 1.9 | 60000 | **114000** |  |
| ESM 10^-13^M |  | 16.4 | 15.4 |  | 1.1 |  |  |  |  |  |
| ESM 10^-14^M |  | 20.2 | 19.0 |  |  |  |  |  | ***MYC*** |  |
| **SM1** | D(-13/-15) | 23.7 | 23.9 | -0.2 | (-1.1) | -1.3 | 2.5 | 600 | **1500** | **13000** |

ISM: internal standards mixture. ESM: external standards mixture. NT Cq: native template quantification cycle. IS Cq: internal standard quantification cycle. ΔCq: quantification cycle difference of NT and IS, NT Cq - IS Cq. mol.: molecules. SM1: surgically removed, formalin-fixed, paraffin-embedded sample 1.

**Figure S1.**


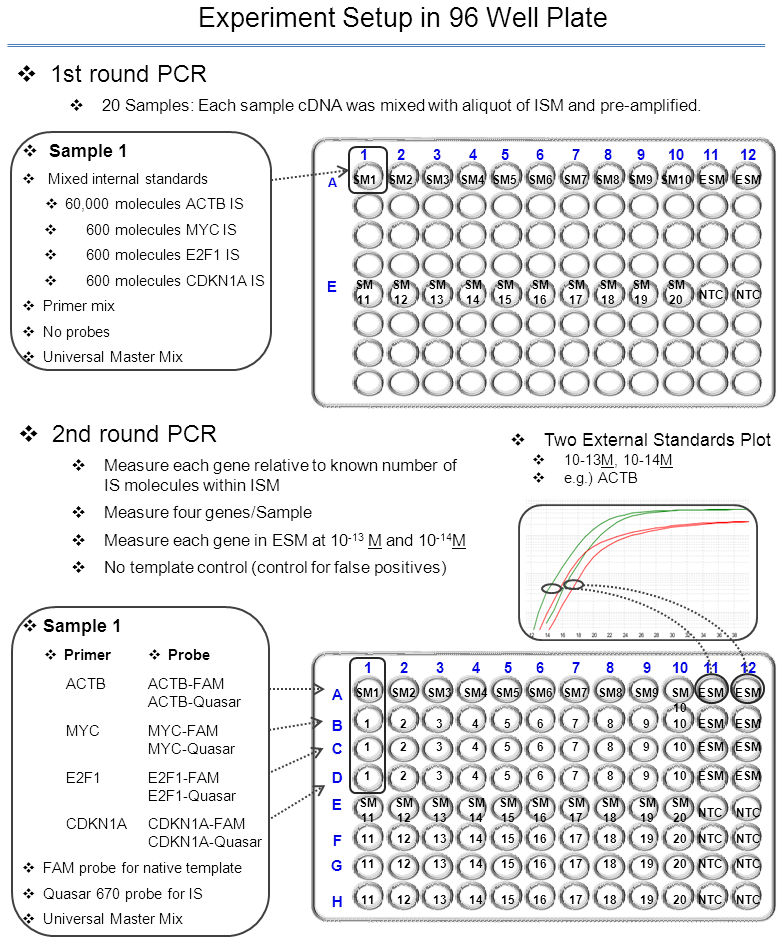


**Figure S2.**


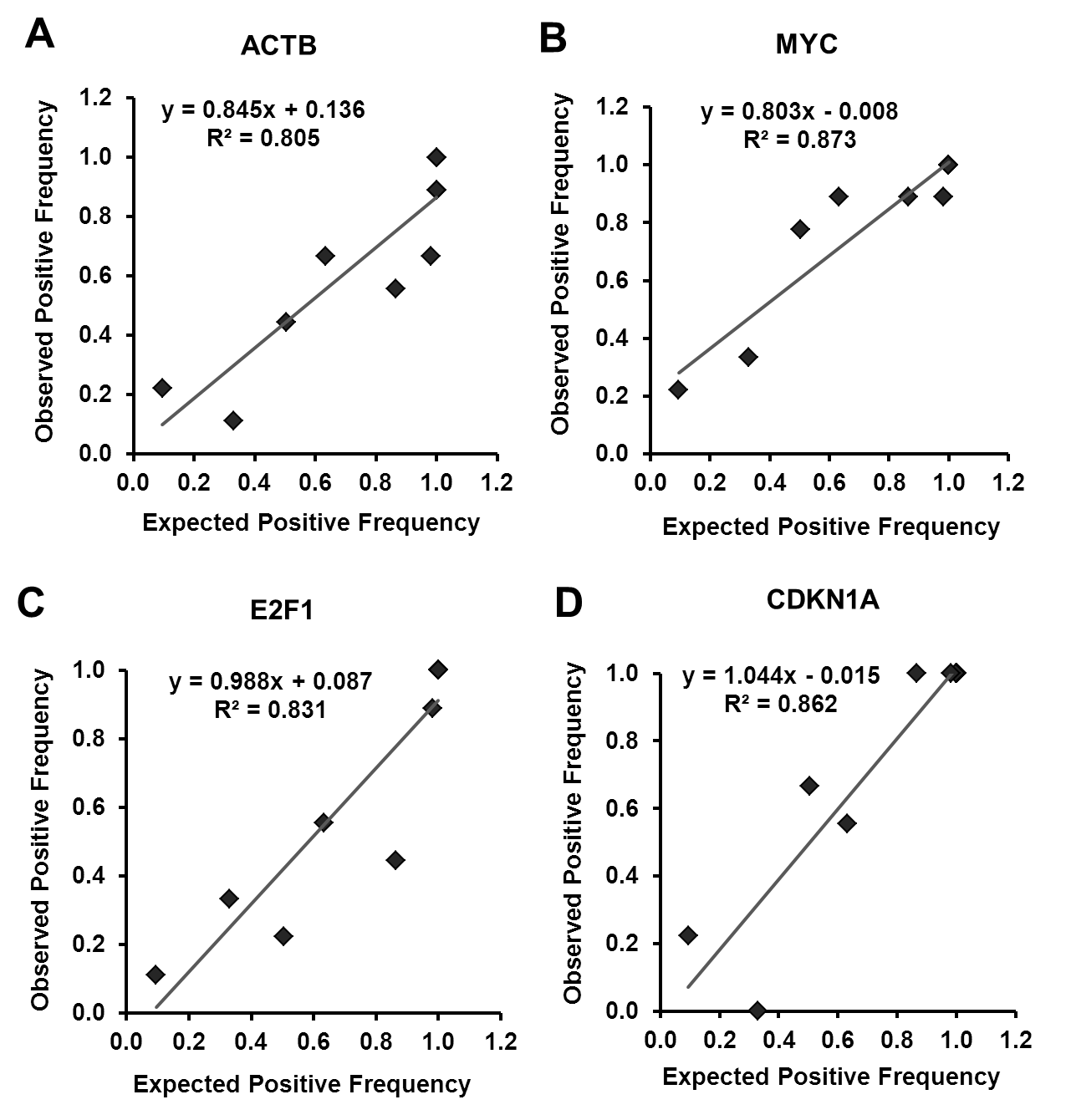


**Figure S3**.


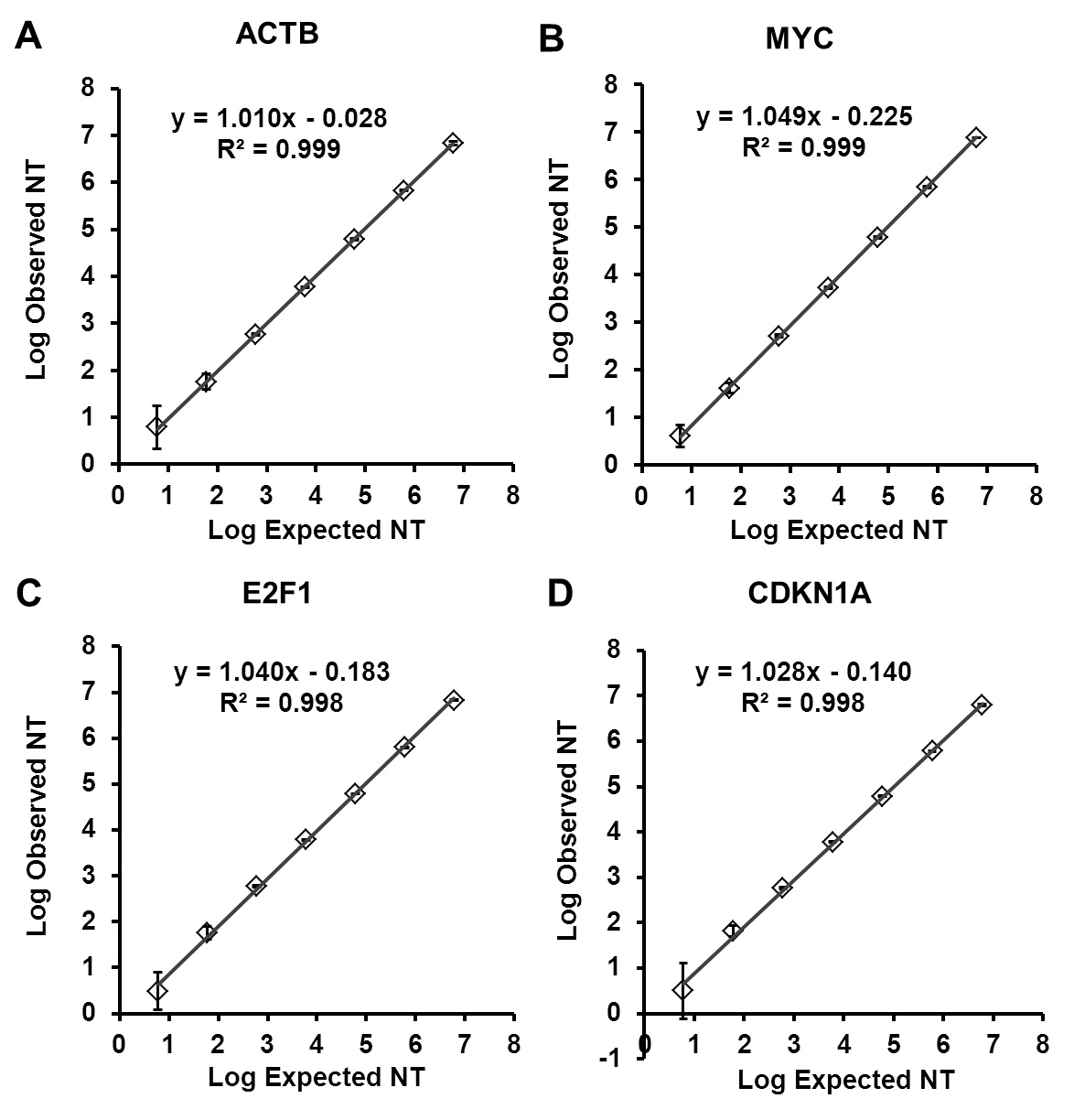


**Figure S4**.


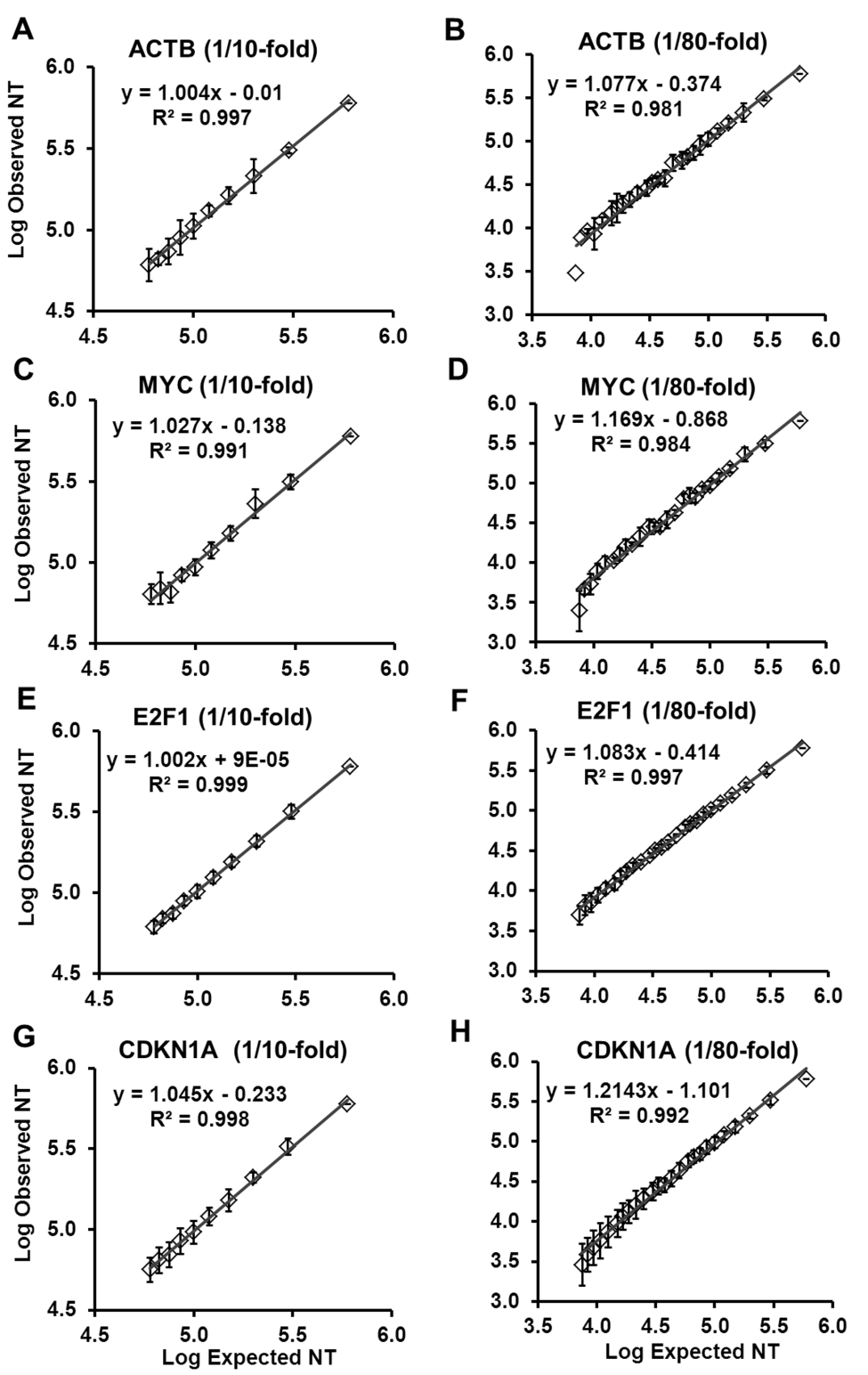


**Figure S5**.


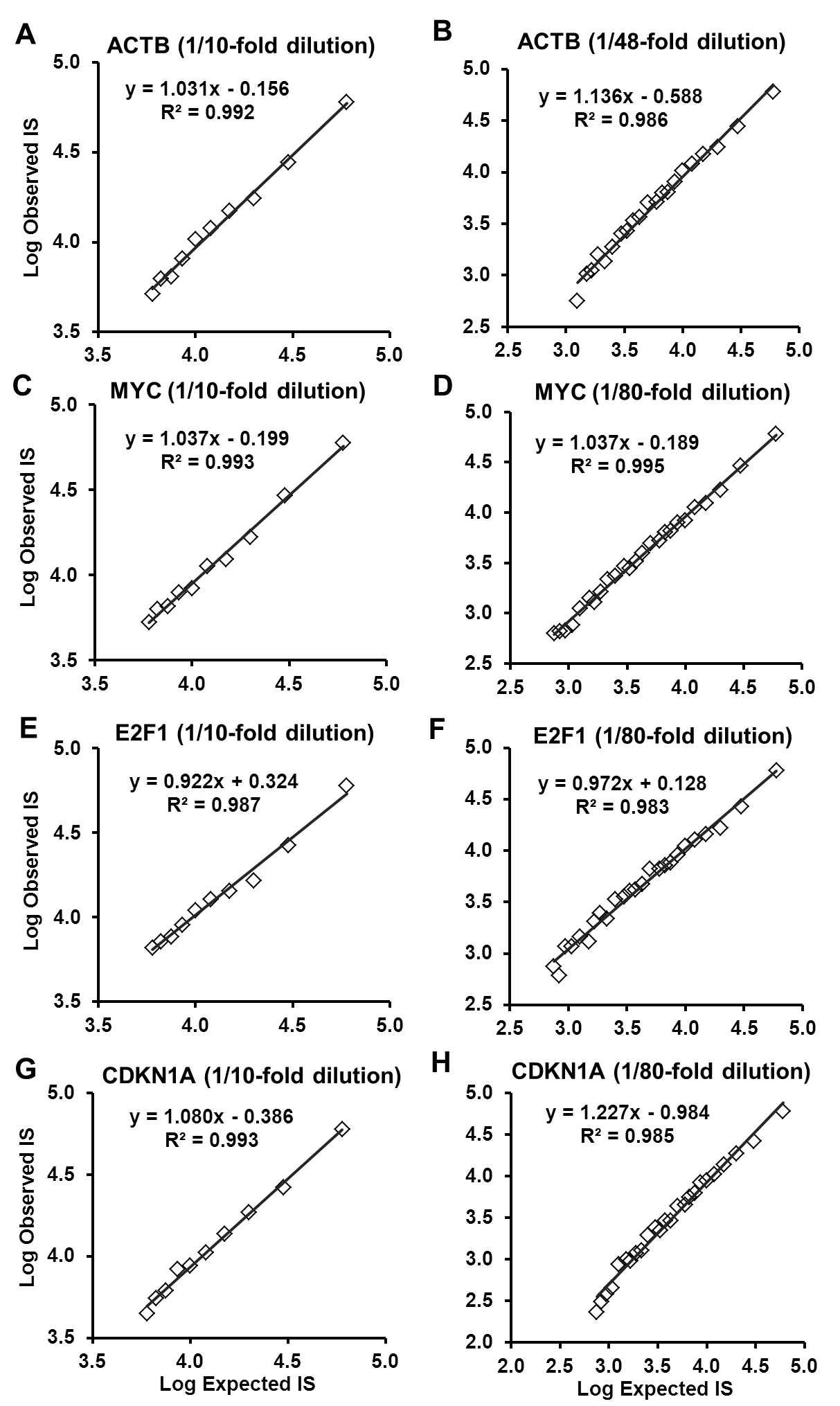


**Table S2**.

| **(A) *ACTB*** | | | | |
| --- | --- | --- | --- | --- |
| **ESM** | **Expected NT** | **Average** | **SD** | **CV** |
| 10^-11^M | 6000000 | 6780000 | 600000 | 0.09 |
| 10^-12^M | 600000 | 671000 | 16300 | 0.02 |
| 10^-13^M | 60000 | 62600 | 2970 | 0.05 |
| 10^-14^M | 6000 | 5880 | 207 | 0.04 |
| 10^-15^M | 600 | 577 | 21 | 0.04 |
| 10^-16^M | 60 | 61 | 26 | 0.42 |
| 10^-17^M | 6 | 9 | 10 | 1.06 |
| **Average of CV from 10^-11^M to 10^-16^M** | | | | **0.11** |
| **Average of CV from 10^-11^M to 10^-17^M** | | | | **0.25** |

| **(B) *MYC*** | | | | |
| --- | --- | --- | --- | --- |
| **ESM** | **Expected NT** | **Average** | **SD** | **CV** |
| 10^-11^M | 6000000 | 7590000 | 105000 | 0.01 |
| 10^-12^M | 600000 | 700000 | 27500 | 0.04 |
| 10^-13^M | 60000 | 61200 | 3190 | 0.05 |
| 10^-14^M | 6000 | 5350 | 194 | 0.04 |
| 10^-15^M | 600 | 515 | 39 | 0.08 |
| 10^-16^M | 60 | 42 | 10 | 0.25 |
| 10^-17^M | 6 | 4 | 2 | 0.43 |
| **Average of CV from 10^-11^M to 10^-16^M** | | | | **0.08** |
| **Average of CV from 10^-11^M to 10^-17^M** | | | | **0.13** |

| **(C) *E2F1*** | | | |  |
| --- | --- | --- | --- | --- |
| **ESM** | **Expected NT** | **Average** | **SD** | **CV** |
| 10^-11^M | 6000000 | 6790000 | 221000 | 0.03 |
| 10^-12^M | 600000 | 635000 | 13400 | 0.02 |
| 10^-13^M | 60000 | 61400 | 1180 | 0.02 |
| 10^-14^M | 6000 | 6100 | 114 | 0.02 |
| 10^-15^M | 600 | 602 | 25 | 0.04 |
| 10^-16^M | 60 | 60 | 20 | 0.34 |
| 10^-17^M | 6 | 4 | 4 | 0.95 |
| **Average of CV from 10^-11^M to 10^-16^M** | | | | **0.08** |
| **Average of CV from 10^-11^M to 10^-17^M** | | | | **0.20** |

| **(D) *CDKN1A*** | | | |  |
| --- | --- | --- | --- | --- |
| **ESM** | **Expected NT** | **Average** | **SD** | **CV** |
| 10^-11^M | 6000000 | 6140000 | 260000 | 0.04 |
| 10^-12^M | 600000 | 599000 | 10600 | 0.02 |
| 10^-13^M | 60000 | 60800 | 1340 | 0.02 |
| 10^-14^M | 6000 | 6000 | 138 | 0.02 |
| 10^-15^M | 600 | 578 | 23 | 0.04 |
| 10^-16^M | 60 | 68 | 20 | 0.29 |
| 10^-17^M | 6 | 6 | 9 | 1.35 |
| **Average of CV from 10^-11^M to 10^-16^M** | | | | **0.07** |
| **Average of CV from 10^-11^M to 10^-17^M** | | | | **0.25** |

ESM: external standards mixture. NT: native template. IS: internal standard. SD: standard deviation. CV: coefficient of variation.

**Table S3**.

| **NT Dilution** | **Expected NT** | **Average** | **SD** | **CV** |
| --- | --- | --- | --- | --- |
| NT 1/1 | 600000 | 600000 | 0 | 0 |
| NT 1/2 | 300000 | 317000 | 27600 | 0.09 |
| NT 1/3 | 200000 | 217000 | 33800 | 0.16 |
| NT 1/4 | 150000 | 156000 | 16800 | 0.11 |
| NT 1/5 | 120000 | 124000 | 11800 | 0.10 |
| NT 1/6 | 100000 | 99700 | 13400 | 0.13 |
| NT 1/7 | 85700 | 87400 | 13300 | 0.15 |
| NT 1/8 | 75000 | 71300 | 9900 | 0.14 |
| NT 1/9 | 66700 | 67400 | 9500 | 0.14 |
| NT 1/10 | 60000 | 61000 | 8800 | 0.14 |
| **Average from 1/1 to 1/10 dilution** | | | | **0.12** |
| NT 1/12 | 50000 | 48000 | 9200 | 0.19 |
| NT 1/14 | 42900 | 37300 | 6900 | 0.19 |
| NT 1/16 | 37500 | 31900 | 5000 | 0.16 |
| NT 1/18 | 33300 | 30600 | 4600 | 0.15 |
| NT 1/20 | 30000 | 27400 | 5200 | 0.19 |
| **Average from 1/1 to 1/20 dilution** | | | | **0.14** |
| NT 1/24 | 25000 | 22500 | 4600 | 0.20 |
| NT 1/28 | 21400 | 19100 | 4200 | 0.22 |
| NT 1/32 | 18800 | 16600 | 3900 | 0.23 |
| NT 1/36 | 16700 | 14600 | 4600 | 0.31 |
| NT 1/40 | 15000 | 12100 | 3700 | 0.31 |
| **Average from 1/1 to 1/40 dilution** | | | | **0.17** |
| NT 1/48 | 12500 | 10200 | 2500 | 0.25 |
| NT 1/56 | 10700 | 8000 | 2700 | 0.34 |
| NT 1/64 | 9380 | 6800 | 2300 | 0.34 |
| NT 1/72 | 8330 | 5700 | 2000 | 0.35 |
| NT 1/80 | 7500 | 3600 | 1600 | 0.45 |
| **Average from 1/1 to 1/80 dilution** | | | | **0.20** |

NT: native template. SD: standard deviation. CV: coefficient of variation.

**Figure S6**.


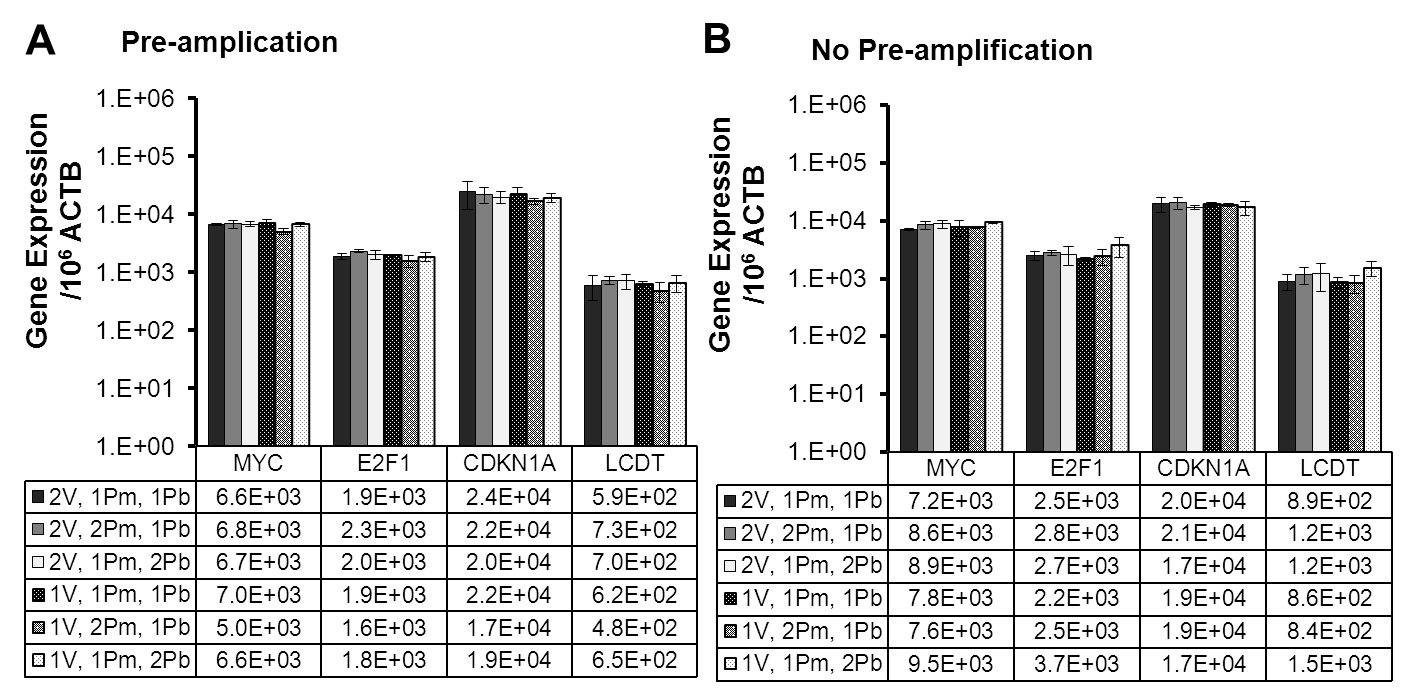


**Table S4**.

|  | Benign |  | Malignant |
| --- | --- | --- | --- |
| SB1 | TB | SM1 | Squamous cell carcinoma |
| SB2 | Emphysema | SM2 | Adenocarcinoma |
| SB3 | UIP | SM3 | Squamous cell carcinoma |
| SB4 | COPD | SM4 | Adenocarcinoma |
| SB5 | Emphysema | SM5 | Squamous cell carcinoma |
| SB6 | UIP | SM6 | Adenocarcinoma |
| SB7 | Foreign body granulomas | SM7 | Adenocarcinoma |
| SB8 | DAD | SM8 | Adenocarcinoma |
| SB9 | Granulomas | SM9 | Squamous cell carcinoma |
| SB10 | Pneumonia | SM10 | Adenocarcinoma |

SB: surgically removed, benign sample. SM: surgically removed, malignant sample. TB: tuberculosis. COPD: chronic obstructive pulmonary disease. UIP: usual interstitial pneumonia. DAD: diffuse alveolar damage.

**Table S5**.

| Benign | RNA (µg) | A260/ 280 | Malignant | RNA (µg) | A260/ 280 |
| --- | --- | --- | --- | --- | --- |
| SB1 | 21.2 | 2.02 | SM1 | 16.1 | 2.00 |
| SB2 | 2.9 | 1.92 | SM2 | 18.1 | 2.00 |
| SB3 | 36.1 | 2.15 | SM3 | 45.8 | 2.04 |
| SB4 | 17.9 | 2.02 | SM4 | 8.4 | 2.02 |
| SB5 | 20.2 | 2.16 | SM5 | 55.4 | 2.13 |
| SB6 | 41.8 | 2.13 | SM6 | 79.7 | 2.09 |
| SB7 | 3.4 | 1.79 | SM7 | 80.3 | 2.03 |
| SB8 | 17.7 | 2.02 | SM8 | 36.3 | 2.05 |
| SB9 | 10.4 | 1.90 | SM9 | 103.4 | 2.03 |
| SB10 | 7.3 | 2.04 | SM10 | 24.6 | 2.00 |
| Average | 17.9 |  | Average | 46.8 |  |

SB: surgically removed, benign sample. SM: surgically removed, malignant sample.
